# Active eosinophils regulate host defence and immune responses in colitis

**DOI:** 10.1038/s41586-022-05628-7

**Published:** 2022-12-12

**Authors:** Alessandra Gurtner, Costanza Borrelli, Ignacio Gonzalez-Perez, Karsten Bach, Ilhan E. Acar, Nicolás G. Núñez, Daniel Crepaz, Kristina Handler, Vivian P. Vu, Atefeh Lafzi, Kristin Stirm, Deeksha Raju, Julia Gschwend, Konrad Basler, Christoph Schneider, Emma Slack, Tomas Valenta, Burkhard Becher, Philippe Krebs, Andreas E. Moor, Isabelle C. Arnold

**Affiliations:** 1 Institute of Experimental Immunology, University of Zürich, Zürich, Switzerland; 2 Department of Biosystems Science and Engineering, ETH Zürich, Basel, Switzerland; 3 Institute of Pathology, University of Bern, Bern, Switzerland; 4 Institute of Molecular Cancer Research, University of Zürich, Zürich, Switzerland; 5 Institute of Physiology, University of Zürich, Zürich, Switzerland; 6 Department of Molecular Life Sciences, University of Zürich, Zürich, Switzerland; 7 Institute for Food, Nutrition and Health, D-HEST, ETH Zürich, Zürich, Switzerland; 8 Botnar Research Center for Child Health, Basel, Switzerland; 9 Institute of Molecular Genetics of the Czech Academy of Sciences, Prague, Czech Republic

**Keywords:** Mucosal immunology, Eosinophils, Gene expression profiling

## Abstract

In the past decade, single-cell transcriptomics has helped to uncover new cell types and states and led to the construction of a cellular compendium of health and disease. Despite this progress, some difficult-to-sequence cells remain absent from tissue atlases. Eosinophils—elusive granulocytes that are implicated in a plethora of human pathologies^1–5^—are among these uncharted cell types. The heterogeneity of eosinophils and the gene programs that underpin their pleiotropic functions remain poorly understood. Here we provide a comprehensive single-cell transcriptomic profiling of mouse eosinophils. We identify an active and a basal population of intestinal eosinophils, which differ in their transcriptome, surface proteome and spatial localization. By means of a genome-wide CRISPR inhibition screen and functional assays, we reveal a mechanism by which interleukin-33 (IL-33) and interferon-γ (IFNγ) induce the accumulation of active eosinophils in the inflamed colon. Active eosinophils are endowed with bactericidal and T cell regulatory activity, and express the co-stimulatory molecules CD80 and PD-L1. Notably, active eosinophils are enriched in the lamina propria of a small cohort of patients with inflammatory bowel disease, and are closely associated with CD4^+^ T cells. Our findings provide insights into the biology of eosinophils and highlight the crucial contribution of this cell type to intestinal homeostasis, immune regulation and host defence. Furthermore, we lay a framework for the characterization of eosinophils in human gastrointestinal diseases.

## Abstract (web summary)

Single-cell transcriptomic profiling and functional assays are used to identify subpopulations of eosinophils that are present in the mouse gastrointestinal tract and provide insight into the role of these cells in inflammatory bowel diseases in humans.

## Main

Eosinophils are granulocytes that reside mainly in the thymus, uterus, lung, adipose tissue and gastrointestinal (GI) tract^1^. Their accumulation is typical of disease states such as allergic airway inflammation, atopic dermatitis, eosinophilic oesophagitis and inflammatory bowel diseases (IBD)^2–5^. GI eosinophils contribute to various homeostatic processes, including preserving the epithelial barrier, supporting tissue architecture, maintaining populations of immune cells and regulating local immune responses^6–9^. However, their function during intestinal inflammation is unclear^10^. Moreover, the presence of functionally distinct eosinophil subsets and their ontogenetic relationship have remained largely uninvestigated owing to technical challenges preventing their transcriptomic interrogation. Indeed, eosinophils are virtually absent from human and mouse single-cell RNA sequencing (scRNA-seq) atlases^11,12^, and thus represent a blind spot in our understanding of cell-type-specific contributions to disease. Here, we fill this gap in knowledge by resolving eosinophil transcriptional and functional heterogeneity along their developmental trajectory from the bone marrow (BM) to tissues of residency, and by defining their role during intestinal inflammation.

## A-Eos and B-Eos are two GI eosinophil subsets

**Fig. 1:**
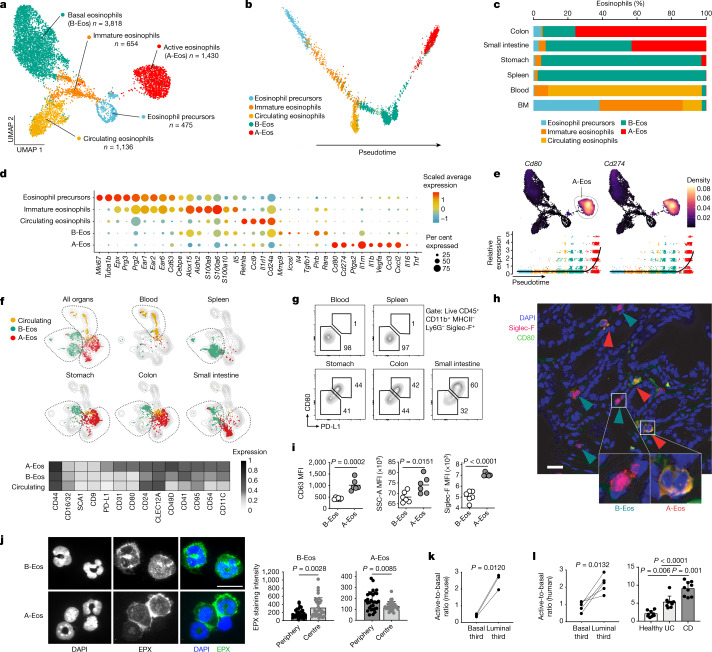
A-Eos and B-Eos are two distinct GI-resident eosinophil subsets. **a**, Uniform manifold approximation and projection (UMAP) of eosinophil transcriptomes obtained from the BM, blood, spleen, small intestine, stomach and colon of *Il5*-tg mice (*n* = 3). **b**, Eosinophil differentiation trajectory. **c**, Subset distribution across organs (% of eosinophils). **d**, Expression of cluster marker genes. A complete list of cluster markers is available in Supplementary Table 1. **e**, Top, UMAP of *Cd80* and *Cd274* expression. Bottom, expression levels over pseudotime. **f**, Top, UMAP of eosinophil proteomic (spectral flow cytometry) profiles isolated from blood, spleen, stomach, colon and small intestine. Bottom, heat map of median surface marker expression across subsets (*n* = 5, B6J). **g**, Representative FACS plots of A-Eos (PD-L1^+^CD80^+^) and PD-L1^−^CD80^−^ eosinophils across organs. Numbers indicate percentage of eosinophils. **h**, Representative immunofluorescence of Siglec-F and CD80 in the mouse colon (*n* = 3, B6J). Arrows mark Siglec-F^+^CD80^+^ A-Eos (red) and Siglec-F^+^CD80^−^ B-Eos (green). Nuclei stained with DAPI. Scale bar, 20 µm. **i**, Mean fluorescence intensity (MFI) of CD63, SSC-A and Siglec-F in colonic A-Eos and B-Eos (*n* = 6, B6J). Medians are shown. Two-tailed unpaired Student’s *t*-test. **j**, Left, representative images of cytospinned intestinal A-Eos and B-Eos stained with anti-EPX and DAPI (*n* = 3, *Il5-*tg). Scale bar, 10 µm. Right, quantification of EPX staining intensity at cell periphery and centre. Data are mean ± s.d. Two-tailed unpaired Student’s *t*-test. **k**, Active-to-basal ratio in luminal versus basal third of colonic crypts (*n* = 3, B6J). Two-tailed paired Student’s *t*-test. **l**, Left, active-to-basal ratio in luminal versus basal third of colonic crypts of healthy human colon cores (*n* = 5). Two-tailed paired Student’s *t*-test. Right, active-to-basal ratio in samples from healthy individuals (5 individuals, 9 cores), patients with Crohn’s disease (CD; 5 individuals, 9 cores) and patients with ulcerative colitis (UC; 4 individuals, 8 cores) samples. One-way ANOVA. Data are mean ± s.d. Patient information is provided in Supplementary Table 2. In **a**,**b**,**e**,**f**, dots represent single cells, coloured by cluster identity.

**Extended Data Fig. 1:**
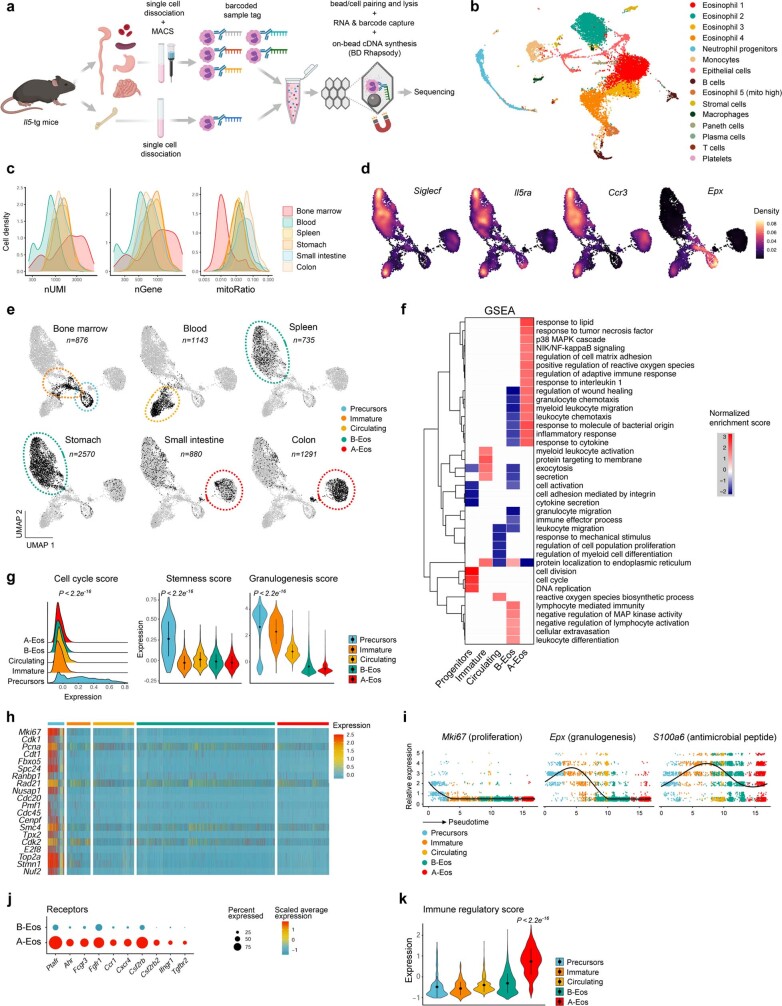
scRNA-seq reveals five distinct eosinophil subpopulations. **a**, Experimental workflow of scRNA-seq. **b**, UMAP of all sequenced single-cell transcriptomes passing quality control, clustered and annotated manually based on marker gene expression. **c**, Distribution of unique molecular identifiers (nUMI, log10 normalized), genes (nGenes, log10 normalized) and mitochondrial gene fraction (mitoRatio, log10 normalized) per cell across samples. **d**, Expression density of canonical eosinophil marker genes. **e**, Subset organ distribution. Dashed lines indicate eosinophil subsets from Fig. 1a. **f**, Significantly enriched (adjusted *P* < *0.05*) GSEA terms across clusters. Kolmogorov–Smirnov test. **g**, Left: cell-cycle score. Middle: stemness score. Right: granulogenesis score. Data represents mean ± SD. Two-sided Wilcoxon test (n = 3, *Il5*-tg). **h**, Expression of cell-cycle genes across eosinophil subsets. Rows are genes and columns are single cells, coloured by scaled expression. **i**, Expression of *mKi67*, *Epx* and *S100a6* over pseudotime. **j**, Receptor gene expression in A-Eos and B-Eos. **k**, Immune-regulatory score across subsets. Data represents mean ± SD. Two-sided Wilcoxon test (n = 3, *Il5*-tg). Genes used for scores and signatures are listed in Supplementary Table 3.

By minimizing shear stress, degranulation and consequent transcript degradation (Extended Data Fig. 1a), we obtained single-cell transcriptomes from eosinophils isolated from the BM, blood, spleen, stomach, small intestine and colon of *Il5*-tg mice, a strain that has high eosinophil counts across tissues^13^ (Extended Data Fig. 1b,c). We found that 89% of all cells widely expressed the bona fide eosinophil markers *Siglecf*, *Il5ra*, *Ccr3* and *Epx* (Extended Data Fig. 1d). Clustering revealed five subpopulations ordered along a developmental trajectory (Fig. 1a,b). Highly cycling precursors and immature eosinophils were primarily present in the BM, and circulating eosinophils were mainly in the blood. Two subsets, termed active eosinophils (A-Eos) and basal eosinophils (B-Eos), populated the GI tissues in varying proportions (Fig. 1c and Extended Data Fig. 1e).

Eosinophil subsets exhibited distinct transcriptional profiles across organs and differed in their cytokine, effector-molecule and receptor repertoire, indicating that they have highly specialized functions (Fig. 1d and Extended Data Fig. 1f). Pseudotime analysis revealed that immature eosinophils downregulate stemness and proliferation programs, and transiently upregulate the expression of granular protein (*Epx*, *Prg2*, *Ear1*, *Ear2* and *Ear6*) and antimicrobial peptide genes (*S100a6*, *S100a9* and *S100a10*) (Extended Data Fig. 1g–i). Circulating eosinophils were characterized by high expression of *Retnla* and of the adhesion protein *Cd24a*, whereas B-Eos expressed effector molecules that are involved in tissue morphogenesis and remodelling, such as *Mmp9* and *Tgfb1* (Fig. 1d). Placed at the end of the differentiation trajectory, A-Eos were only found in organs of the GI tract and specifically expressed genes encoding multiple bioactive factors (*Il16*, *Tnf*, *Il1b*, *Ccl3*, *Cxcl2*, *Vegfa* and *Ptgs2*) and receptors (*Il1rn*, *Csf2rb*, *Tgfbr2*, *Ccr1*, *Cxcr4*, *Ptafr* and *Ahr*) (Fig. 1d and Extended Data Fig. 1j). Moreover, their expression of the co-stimulatory molecules *Cd80* and *Cd274* (PD-L1) suggests that A-Eos are involved in immune modulation (Fig. 1e and Extended Data Fig. 1k). We thus focused our attention on this subset.

**Extended Data Fig. 2:**
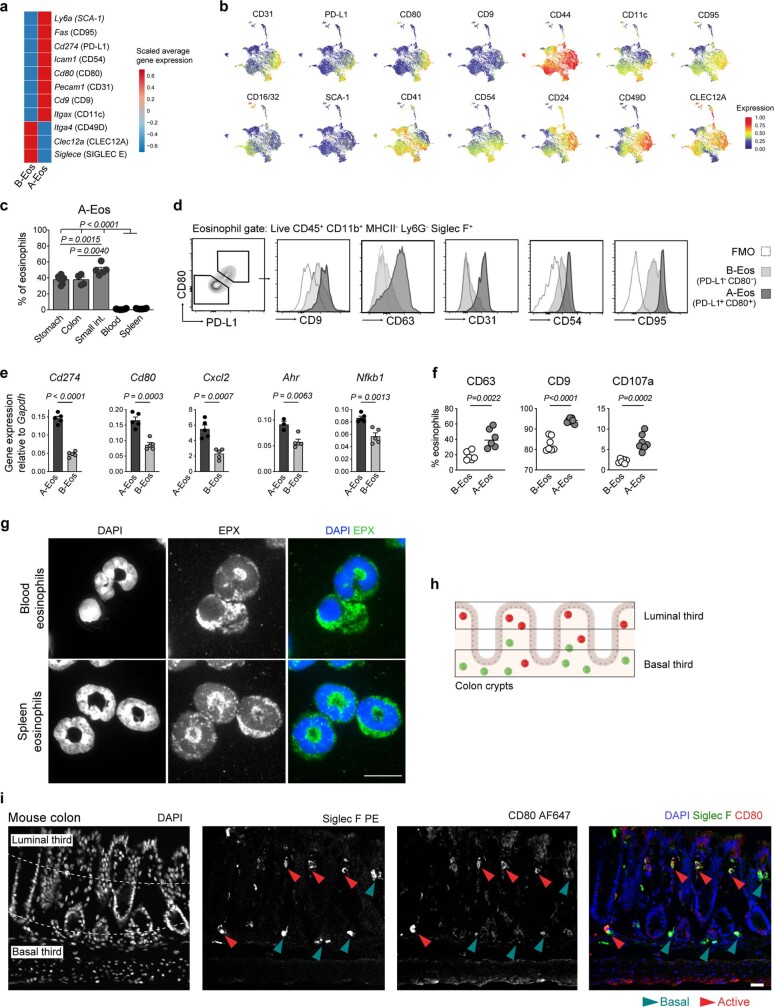
PD-L1 and CD80 expression define active eosinophils in the GI tract. **a**, GI surface marker gene expression in A-Eos and B-Eos. **b**, UMAP showing the normalized protein expression intensity of eosinophil surface markers (n = 4, B6J). **c**, Frequencies of A-Eos as assessed by flow cytometry (n = 4–6, B6J). Data represents mean ± SEM. One-way ANOVA. Data pooled from two independent experiments. **d**, Mean fluorescence intensity (MFI) of CD9, CD31, CD54 and CD95 across colonic eosinophil subsets. FMO: fluorescence minus one. **e**, Expression of A-Eos markers, normalized to *Gapdh* in A-Eos and B-Eos sorted from the small intestine (n = 4, *Il5*-tg). Data represents mean ± SEM. Two-tailed unpaired Student’s *t*-test. **f**, Frequencies of CD63^+^, CD9^+^ and CD107a^+^ cells in A-Eos and B-Eos as assessed by flow cytometry (n = 6-7, B6J). Data represents mean ± SEM. Two-tailed unpaired Student’s *t*-test. Data pooled from two independent experiments. **g**, EPX immunofluorescence in sorted blood and spleen eosinophils (n = 3, *Il5*-tg). Nuclei are stained with DAPI. Scale bar, 10 µm. **h**, Schematic representation of basal (lower) and luminal (upper) third of the mucosa. **i**, Representative immunofluorescence images of Siglec-F and CD80 in the mouse colon (n = 3, B6J). Arrows mark Siglec-F^+^CD80^+^ A-Eos (red) and Siglec-F^+^CD80^−^ B-Eos (green). Nuclei are stained with DAPI. Dashed lines delimit the border of luminal and basal third. Scale bar, 20 µm.

**Extended Data Fig. 3:**
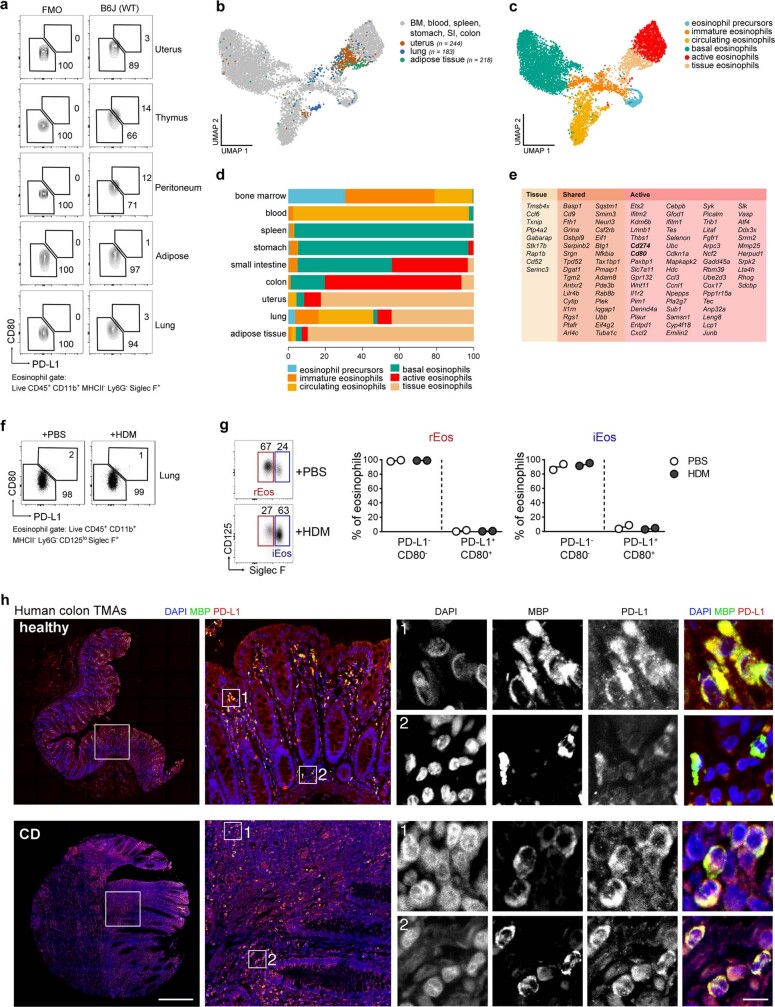
PD-L1^+^CD80^+^ A-Eos are specific to the mouse GI and enriched in human IBD. **a**, Representative FACS plots of PD-L1^+^CD80^+^ and PD-L1^−^ CD80^−^ eosinophils (n = 3, B6J). Numbers indicate % of eosinophils. **b**,**c**, UMAP of eosinophil transcriptomes (shown in Fig. 1a) including those isolated from uterus, lung and adipose tissue (n = 4, *Il5*-tg). Cells coloured by organ (**b**) and by cluster (**c**). **d**, Subset distribution across organs (% of eosinophils). **e**, List of shared or unique markers (logFC > 0.5, *P* adjusted < 0.05) between A-Eos and tissue eosinophils. Non-parametric Wilcoxon rank sum test (FindMarkers function in Seurat). **f**, Representative FACS plots of PD-L1^+^CD80^+^and PD-L1^−^CD80^−^ eosinophils in HDM- or PBS-treated mice (n = 2, B6J). Numbers indicate % of eosinophils. **g**, Left: Gating strategy used to identify resident (rEos) and inflammatory (iEos) eosinophils as described by^17^. Right: quantification of PD-L1^+^CD80^+^ and PD-L1^−^CD80^−^ eosinophils in rEos and iEos. Medians are shown. **h**, MBP and PD-L1 immunofluorescence staining of human tissue microarrays. Representative cores from a healthy individual and a patient with Crohn’s disease are shown (n = 5). Scale bars, 500 µm (core overview) and 10 µm (high magnification insets).

We profiled the surface proteome of blood, small intestine and colon eosinophils in B6J (wild-type) mice by spectral flow cytometry (fluorescence-activated cell sorting (FACS)) and found that the expression of PD-L1 and CD80 was sufficient to identify A-Eos (Fig. 1f–h and Extended Data Fig. 2a–c). PD-L1^+^CD80^+^ cells expressed A-Eos markers at the protein and RNA levels (Extended Data Fig. 2d,e), and exhibited higher secretory activity^14–16^ (CD63, CD9 and CD107a), granularity (SSC-A) and activation (Siglec-F) relative to B-Eos (Fig. 1i and Extended Data Fig. 2f). A-Eos also showed a peripheral distribution of eosinophil peroxidase (EPX), whereas granule localization in B-Eos, splenic and blood eosinophils was more cytosolic (Fig. 1j and Extended Data Fig. 2g). Of note, A-Eos and B-Eos differed in their spatial localization within the colonic mucosa, indicating exposure to and interactions with distinct cellular microenvironments: A-Eos were found significantly closer to the luminal extremity (luminal third), whereas B-Eos were retained near the submucosa (basal third) (Fig. 1k and Extended Data Fig. 2h,i). The presence of A-Eos was restricted to the GI tract, as PD-L1^+^CD80^+^ eosinophils were not found by FACS (Extended Data Fig. 3a) or scRNA-seq (Extended Data Fig. 3b–e) in other tissues in which eosinophils reside, such as the uterus and adipose, and were only detected in small percentages in the thymus and peritoneum. A-Eos further differed from previously reported lung-resident populations and from inflammatory eosinophils recruited during a house dust mite (HDM) airway challenge^17^ (Extended Data Fig. 3f,g).

We next wondered whether A-Eos and B-Eos could also be found in the human GI tract, and whether their proportions are affected by colitis. We therefore subjected colon tissue microarrays (TMAs) from healthy individuals and from patients with IBD to major basic protein (MBP) and PD-L1 immunofluorescence analysis (Extended Data Fig. 3h). Similar to our observations in mice, MBP^+^PD-L1^+^ A-Eos were found closer to the lumen than MBP^+^PD-L1^−^ B-Eos, indicating phenotypic correspondence (Fig. 1l). Notably, the relative abundance of A-Eos (active-to-basal ratio) was twofold enriched in samples from patients with ulcerative colitis and fivefold enriched in samples from patients with Crohn’s disease, relative to healthy control individuals (Fig. 1l). This prompted us to investigate the role of A-Eos during intestinal inflammation.

## A-Eos have antibacterial and regulatory functions

**Fig. 2:**
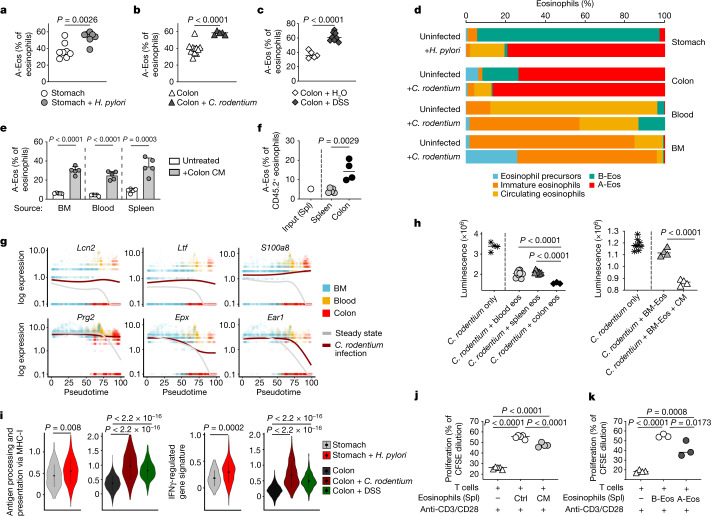
A-Eos have antibacterial and immune-regulatory functions. **a**–**c**, A-Eos frequencies in *H. pylori*-infected (**a**; stomach, *n* = 6), *C. rodentium-*infected (**b**; colon, *n* = 5) and DSS-treated (**c**; colon, *n* = 8) mice relative to uninfected controls (*n* = 5–10, B6J). **a**,**b**, Data are pooled from two independent experiments. Medians are shown. Two-tailed unpaired Student’s *t*-test. **d**, Percentage of eosinophil subsets across organs at steady state and during infection, as assessed by scRNA-seq. **e**, A-Eos frequencies after conditioning with colon CM. Input: BM-derived (*n* = 5, B6J), blood (*n* = 5, *Il5*-tg) and splenic (*n* = 5, *Il5*-tg) eosinophils. Data are mean ± s.d. Two-tailed unpaired Student’s *t*-test. **f**, A-Eos frequencies among adoptively transferred CD45.2^+^ eosinophils in colon and spleen of host, 42 h after injection (*n* = 4, CD45.1). Input A-Eos frequency shown as a reference (spl, splenic eosinophils, *n* = 2, *Il5*-tg). Medians are shown. Two-tailed unpaired Student’s *t*-test. **g**, Gene expression over common pseudotime at steady state (grey) and during *C. rodentium* infection (dark red). Dots indicate single cells, coloured by organ (BM, blood and colon). **h**, *C. rodentium* (ICC180) viability after exposure to blood, splenic or colonic eosinophils (*n* = 3, pooled *Il5*-tg) or conditioned BM-Eos (*n* = 3, pooled B6J). Technical replicates and medians are shown. Two-tailed unpaired Student’s *t*-test. **i**, Expression of MHC-I-restricted antigen processing and presentation signature and IFNγ-regulated genes. Genes used for scores and signatures are listed in Supplementary Table 3. Data are mean ± s.d. Two-sided Wilcoxon test (*n* = 3, *Il5*-tg). **j**,**k**, Proliferation of anti-CD3 and anti-CD28 (anti-CD3/CD28)-activated, carboxyfluorescein succinimidyl ester (CFSE)-labelled naive CD4^+^ T cells co-cultured with conditioned splenic (**j**; Spl) or sorted GI (**k**) A-Eos and B-Eos (*n* = 7, *Il5*-tg mice). Medians are shown. One-way ANOVA.

**Extended Data Fig. 4:**
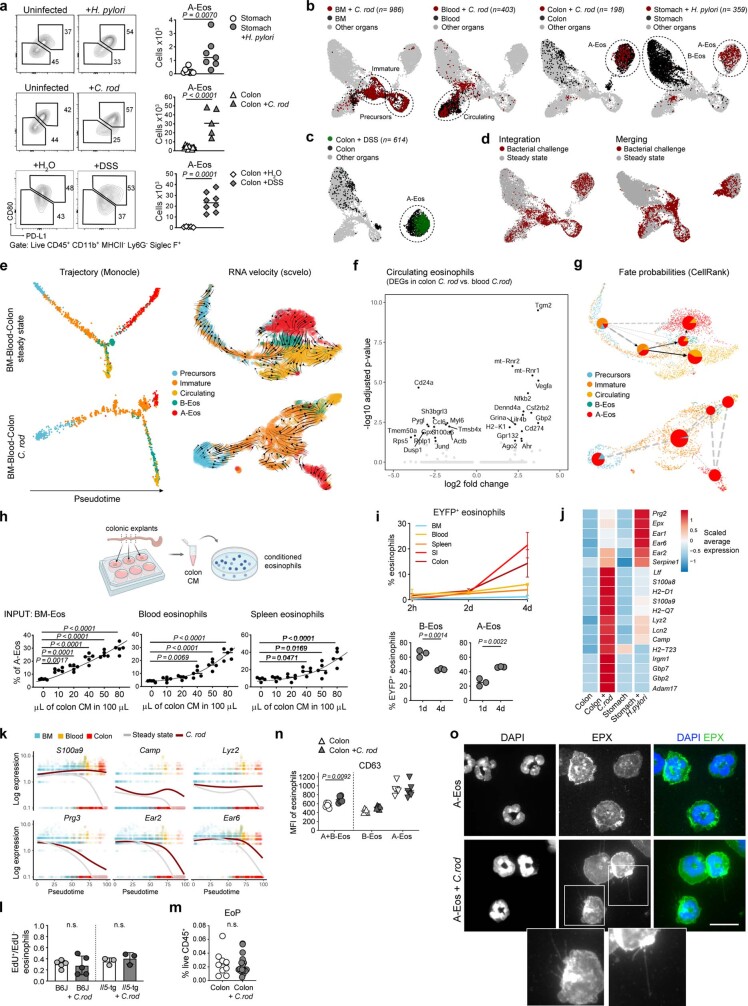
Challenge infection induces a compositional shift toward the A-Eos cluster. **a**, Left: Representative FACS plots of the A-Eos and PD-L1^−^ CD80^−^ eosinophils. Numbers indicate % of eosinophils. Right: Absolute counts of A-Eos of mice shown in Fig. 2a–c. Medians are shown. Two-tailed unpaired Student’s *t*-test. **b**, Data integration of challenge datasets (dark-red dots, n = 4, *Il5*-tg). Steady-state dataset (grey) used as a reference. Corresponding steady-state organs shown in black. **c**, Data integration of DSS dataset (dark-green dots, n = 3, B6J). Steady-state dataset (grey) is used as a reference. Sstate colon shown in black. **d**, UMAP of integrated (left) and merged (right) steady-state (grey) and challenge (red dots) datasets. **e**, Left: BM–blood-colon eosinophil Monocle trajectory at steady state and following *C. rodentium* infection. Each dot represents a single cell coloured by cluster identity. Right: RNA velocities (scvelo) in BM, blood and colon dataset as steady state and during *C. rodentium* infection. **f**, Significant DEGs (logFC > 0.5, adjusted *P* < 0.05) of circulating eosinophils found in the colon vs in the blood of *C. rodentium*-infected mice. Non-parametric Wilcoxon rank sum test (FindMarkers function in Seurat). **g**, Single-cell fate probabilities as calculated by CellRank and summarized for each cluster as a pie chart. Arrows represent velocity flow. Cells and pie charts coloured by cluster identity. **h**, Top: Workflow of *in vitro* conditioning. Bottom: A-Eos frequencies after conditioning with increasing doses of colon CM. Input: BM-derived (n = 5, B6J), blood (n = 5, *Il5*-tg) and splenic (n = 5, *Il5*-tg) eosinophils. Medians are shown. One-way ANOVA. **i**, Left: EYFP^+^ eosinophil frequencies over time across organs after single tamoxifen pulse in *Id2*^*CreERT2*^*;Rosa*^EYFP^ mice. Data represents mean ± SD. Right: Frequency of A-Eos and B-Eos in colonic EYFP^+^ eosinophils at day 2 and 4 post tamoxifen injection (n = 3, *Id2*^*CreER*^^*T2*^*;Rosa*^*EYFP*^). Medians are shown. Two-tailed unpaired Student’s *t*-test. **j**, Antimicrobial and granulogenesis signature expression in A-Eos. **k**, Gene expression over common pseudotime at steady state (grey) and upon *C. rodentium* infection (dark red). Dots indicate single cells, coloured by organ: BM (blue), blood (yellow) and colon (red). **l**, Edu^+^/Edu^−^ eosinophil ratio in the colon of *C. rodentium*-infected and control B6J (n = 5) and *Il5-*tg (n = 3) mice at day 4 post EdU injection. Data represent mean ± SEM. Two-tailed unpaired Student’s *t*-test. **m**, Frequencies of eosinophil progenitors (gated as Live CD45^+^CD11b^+^IL5Ra^+^Lin-Sca1^−^CD34^+^) in *C. rodentium*-infected (n = 17) and control (n = 9) B6J mice. Medians are shown. Data pooled from two independent experiments. Two-tailed unpaired Student’s *t*-test. **n**, MFI of CD63 in colonic A-Eos and B-Eos of *C. rodentium*-infected and control mice (n = 6, B6J). Medians are shown. Two-tailed unpaired Student’s *t*-test. **o**, EPX immunofluorescence of sorted A-Eos of *C. rodentium*-infected and control mice (n = 5, *Il5-*tg). Nuclei are stained with DAPI. Insets show protrusions. Scale bar, 10 µm.

To assess how local insults affect the dynamics of the eosinophil subsets, we evaluated the frequency of PD-L1^+^CD80^+^ A-Eos in three distinct experimental models of GI inflammation: acute *Citrobacter rodentium* infection in the colon, chronic *Helicobacter pylori* infection in the stomach and dextran sulfate sodium (DSS)-induced colitis (Fig. 2a–c and Extended Data Fig. 4a). A-Eos frequencies and numbers were significantly enriched across all models, reflecting what was observed in IBD and indicating that an increase in the active-to-basal ratio is a general response to epithelial damage and inflammation in the human and mouse gut.

To investigate the subset-specific transcriptional changes that occur during inflammation, we profiled eosinophils from the BM, blood and colon of *C. rodentium*-infected and from the stomach of *H. pylori-*infected *Il5*-tg mice by scRNA-seq (Extended Data Fig. 4b). We also retrieved eosinophil transcriptomes from an independent dataset of magnetically-enriched colonic CD45^+^ cells of DSS-treated B6J (wild-type) mice^18^ (Extended Data Fig. 4c). These single-cell profiles were integrated in the steady-state transcriptional embedding and mapped with high confidence to the existing clusters. Of note, merging the steady-state and the challenge datasets did not reveal novel inflammation-specific clusters (Extended Data Fig. 4d).

Infection strongly increased the active-to-basal ratio of eosinophils in the colon and stomach, and led to the accumulation of circulating eosinophils within infected tissues (Fig. 2d). Bacterial challenge further induced a relative expansion of immature eosinophils in the blood and BM. Core eosinophil populations are thus maintained during inflammation, but their proportions across organs vary to maximize the production of A-Eos at sites of infection. This compositional shift suggests alterations in the eosinophil differentiation path. Indeed, trajectory inference (Monocle; ref. ^19^) and RNA velocity analysis (scvelo; ref. ^20^) of BM, blood and colon eosinophils during *C. rodentium* infection placed A-Eos as originating directly from immature eosinophils—rather than from B-Eos, as observed at steady state (Extended Data Fig. 4e). Furthermore, circulating eosinophils found in the colon, but not in the blood, of *C. rodentium*-infected mice expressed multiple A-Eos markers, suggesting a bypassing of the B-Eos maturation stage and rapid transition into A-Eos in situ (Extended Data Fig. 4f). Notably, single-cell fate probabilities computed with CellRank (ref. ^21^) defined A-Eos as the major predicted terminal state for all eosinophil subsets, both at steady state and particularly during infection (Extended Data Fig. 4g). This suggests that B-Eos and circulating eosinophils are not alternative end states, but rather differentiation intermediates. In line with this, after in vitro exposure to colon supernatant (conditioned medium (CM)), eosinophils that differentiated from the BM (BM-Eos; mostly precursors and immature eosinophils), or that were derived from the blood (mainly circulating eosinophils) or spleen (mainly B-Eos), all equally acquired PD-L1^+^ and CD80^+^ surface expression in a dose-dependent manner, indicating that the potential of eosinophils to differentiate into A-Eos is maintained throughout their maturation (Fig. 2e and Extended Data Fig. 4h). We performed genetic fate mapping in *Id2*^*CreERT2*^*;Rosa*^*EYPF*^ mice, a reporter strain in which Id2-Cre-expressing cells are inducibly labelled with EYFP. After a single tamoxifen pulse, the frequencies of colonic B-Eos among EYFP^+^ eosinophils decreased over time, whereas A-Eos frequencies increased, suggesting the conversion of B-Eos to A-Eos in vivo (Extended Data Fig. 4i). Similarly, adoptively transferred CD45.2 splenic eosinophils (B-Eos) migrated into the colon of CD45.1 hosts and showed evidence of in situ maturation into A-Eos (Fig. 2f). Cumulatively, these data suggest lineage plasticity and sequential ontogeny, with circulating eosinophils and B-Eos as metastable transition states along a dynamic differentiation continuum that culminates with A-Eos.

To investigate the transcriptional changes that are elicited by infection along the eosinophil maturation continuum, we aligned BM–blood–colon trajectories during steady state and *C. rodentium* infection to a common pseudotime axis^22^. At steady state, the expression of genes that encode granular proteins and antimicrobial peptides was only transiently upregulated by precursors and immature eosinophils, and therefore restricted to the BM; by contrast, infection induced the sustained expression of granulogenesis and antimicrobial gene programs in circulating and colonic A-Eos (Fig. 2g and Extended Data Fig. 4j,k). Notably, this did not result from altered recruitment kinetics, as assessed by 5-ethynyl-2'-deoxyuridine (EdU) pulsing, or from extramedullary haematopoiesis, as no lineage-committed progenitors (IL-5-Rα^+^Lin^−^Sca1^−^CD34^+^) were detected in the colon afterinfection (Extended Data Fig. 4l,m). Moreover, the expression of CD63 in A-Eos was unaltered by bacterial challenge, indicating that the net increase in the levels of CD63 results from the accumulation of A-Eos rather than their enhanced secretory activity (Extended Data Fig. 4n). However, colonic A-Eos exhibited a marked change in morphology after *C. rodentium* infection, with evidence of cellular protrusions resembling extracellular DNA traps at sites of peripheral EPX accumulation (Extended Data Fig. 4o). We previously reported impaired bacterial clearance and enhanced colonic immunopathology in *C. rodentium-*infected eosinophil-deficient mice^23^. Hence, we assessed the bactericidal potential of A-Eos in co-culture with a bioluminescent *C. rodentium* strain. Colonic eosinophils (mainly A-Eos), as well as conditioned BM-Eos, exhibited significantly greater bactericidal activity with respect to blood (circulating), spleen (B-Eos) or unconditioned BM-Eos (immature eosinophils) (Fig. 2h). Our data therefore suggest that A-Eos are a highly specialized subset involved in bacterial control and endowed with antimicrobial and cytotoxic properties.

**Extended Data Fig. 5:**
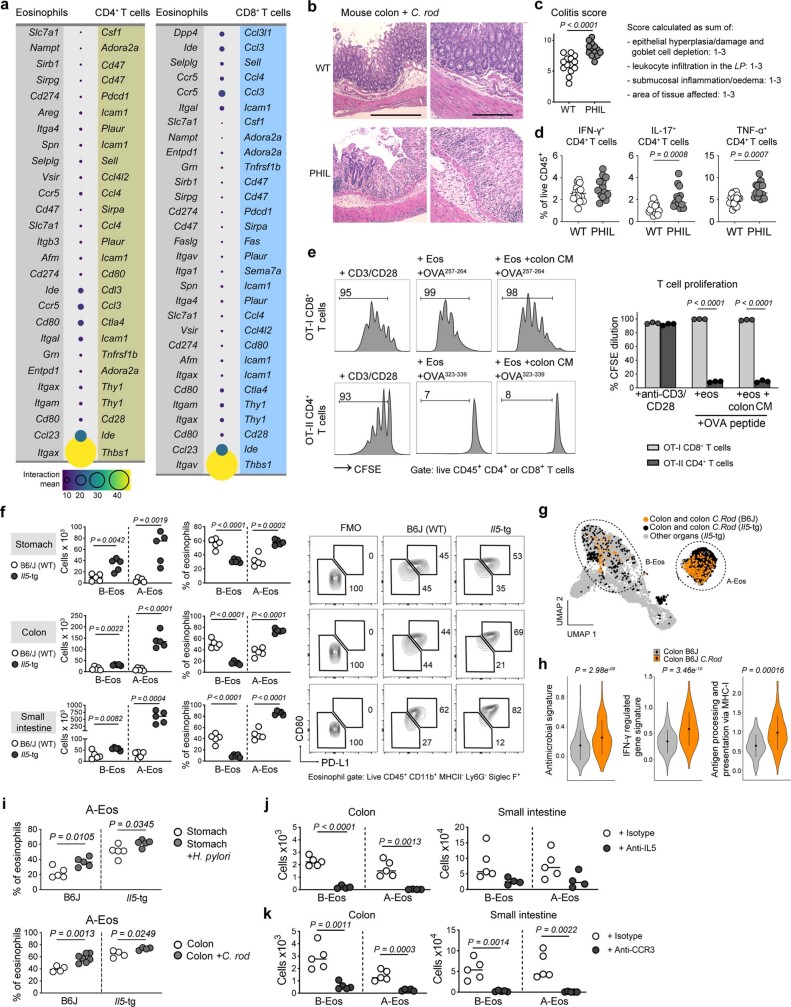
A-Eos interact with T cells. **a**, Ligand–receptor interactions between eosinophils and CD4^+^ T cells (left) or CD8^+^ T cells (right) predicted by CellPhoneDB. Dot size and colour indicate interaction mean. **b**,**c**, Representative H&E-stained colonic sections (**b**) and colitis score (**c**) in B6J (n = 17) and PHIL (n = 13) mice assessed by histopathological examination; data are pooled from two independent experiments. Medians are shown. Two-tailed unpaired Student’s *t*-test. Scale bars, 100 µm. **d**, Frequencies of IFNγ, IL-17 and TNF-expressing colonic CD4^+^ T cells of DSS-treated B6J (n = 17) and PHIL (n = 13) mice. Medians are shown. Two-tailed unpaired Student’s *t*-test. **e**, Left: CFSE dilution of T cells co-cultured with BM-derived eosinophils conditioned as indicated and loaded with ovalbumin (OVA) peptide. Right: Representative FACS plots of the CFSE dilution. Numbers indicate % of CFSE dilution (n = 3, B6J). Data represents mean ± SEM. Two-tailed unpaired Student’s *t*-test. **f**, Left: A-Eos (PD-L1^+^CD80^+^) and B-Eos (PD-L1^−^CD80^−^) frequencies in stomach, colon and small intestine of B6J (n = 5) and *Il5*-tg (n = 5) mice. Medians are shown. Two-tailed unpaired Student’s *t*-test. Right: Representative FACS plots. Numbers indicate % of eosinophils. **g**, UMAP of B6J colonic eosinophils (orange) at steady state (n = 6) and during *C. rodentium* infection (n = 5) integrated in the *Il5*-tg dataset (grey). *Il5*-tg colonic eosinophils at steady state and during *C. rodentium* infection in black. **h**, Antimicrobial signature, IFNγ-regulated gene signature and antigen processing and presentation via MHC-I in B6J colon and B6J colon + *C. rodentium*. Data represents mean ± SD. Two-sided Wilcoxon test (n = 3). **i**, A-Eos frequencies in *H. pylori*-infected (stomach, n = 5) and *C. rodentium*-infected (colon, n = 4–7) B6J and *Il5*-tg mice, relative to uninfected controls. Medians are shown. Two-tailed unpaired Student’s *t*-test. **j**,**k** Absolute counts of A-Eos and B-Eos in colon and small intestine of B6J mice treated with anti-IL-5 (**j**, n = 5) or anti-CCR3 (**k**, n = 5) neutralizing antibodies and the respective isotype control. Medians are shown. Two-tailed unpaired Student’s *t*-test.

Across all our inflammation models, A-Eos specifically upregulated gene sets that are involved in immune modulation, IFNγ signalling and MHC-I-restricted antigen processing and presentation (Fig. 2i). Moreover, CellPhoneDB (ref. ^24^) identified numerous potentially interacting ligand–receptor pairs between A-Eos, CD4^+^ and CD8^+^ T cells (Extended Data Fig. 5a). After treatment with DSS, eosinophil-deficient (PHIL) mice exhibited increased colitis severity (Extended Data Fig. 5b,c) and stronger T helper 17 cell (T_H_17) responses relative to their wild-type littermates, as well as increased production of TNF and IFNγ by CD4^+^ T cells^25^ (Extended Data Fig. 5d). These data corroborate our previous report of an immune-regulatory role of eosinophils^23^, which, given their relative abundance and specific expression of co-stimulatory molecules, may be attributed to A-Eos. Co-culture of both conditioned and unconditioned BM-Eos with OT-I CD8^+^ T cells, but not OT-II CD4^+^ T cells, resulted in robust T cell proliferation in an antigen-dependent manner, suggesting that eosinophils can present antigen via MHC-I and TCR interactions (Extended Data Fig. 5e). Conversely, conditioning of BM-Eos into A-Eos was required for the downregulation of CD4^+^ T cell proliferation after anti-CD3- and anti-CD28-mediated stimulation (Fig. 2j). Indeed, only sorted intestinal A-Eos, and not B-Eos, were able to inhibit the proliferation of CD4^+^ T cells (Fig. 2k), suggesting that this subset attenuates CD4^+^ T cell responses during inflammation^9,23,26^.

Of note, as IL-5 is a known driver of eosinophil maturation and survival^27^, we conducted comparative flow cytometry and scRNA-seq analyses between B6J and *Il5*-tg mice. Aside from higher steady-state frequencies of A-Eos in *Il5*-tg mice (Extended Data Fig. 5f), we did not detect any transgene-specific effects during challenge (Extended Data Fig. 5g–i). Moreover, both subsets were similarly affected by anti-IL-5 treatment and equally depended on eotaxin–CCR3 interactions for their GI tissue accumulation (Extended Data Fig. 5j,k).

## A-Eos maturation is induced locally by IL-33

**Fig. 3:**
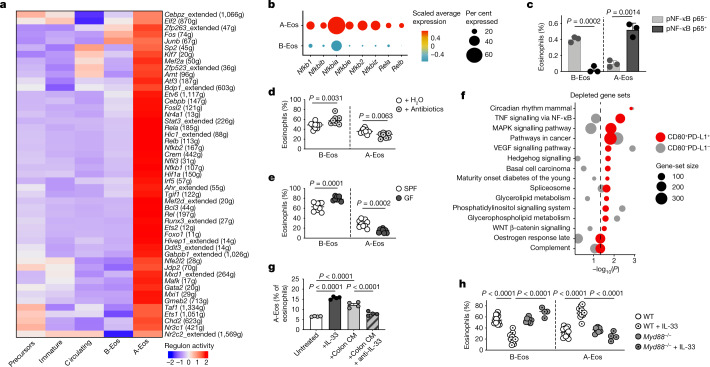
A-Eos maturation is induced locally by IL-33. **a**, Activity of A-Eos-specific regulons across clusters. g, number of genes in regulon. **b**, Expression of NF-κB signalling components. **c**, Quantification of pNF-κB p65^+^ cells in colonic A-Eos and B-Eos (*n* = 3, B6J). Data are mean ± s.e.m. Two-tailed unpaired Student’s *t*-test. **d**,**e**, A-Eos and B-Eos frequencies in antibiotic-treated (**d**) (*n* = 16, B6J) and germ-free (GF) (**e**) (*n* = 9, B6J) mice relative to controls. SPF, specific pathogen free. **d**, Data are pooled from two independent experiments. Medians are shown. Two-tailed unpaired Student’s *t*-test. **f**, Depleted gene sets in PD-L1^+^CD80^+^ A-Eos (red) and PD-L1^−^CD80^−^ eosinophils (grey), relative to BM stem cells. Kolmogorov–Smirnov test. Dot size indicates gene-set size. Dashed line indicates *P* = 0.05. **g**, A-Eos frequencies after conditioning of BM-Eos with IL-33, colon CM and anti-IL-33 (*n* = 2, pooled B6J). Technical replicates and mean ± s.e.m. are shown. One-way ANOVA. **h**, Colonic A-Eos and B-Eos frequencies in B6J (*n* = 21) and *Myd88*^−/−^ (*n* = 15) mice treated with IL-33, relative to untreated controls. Medians are shown. Two-tailed unpaired Student’s *t*-test. WT, wild type.

**Extended Data Fig. 6:**
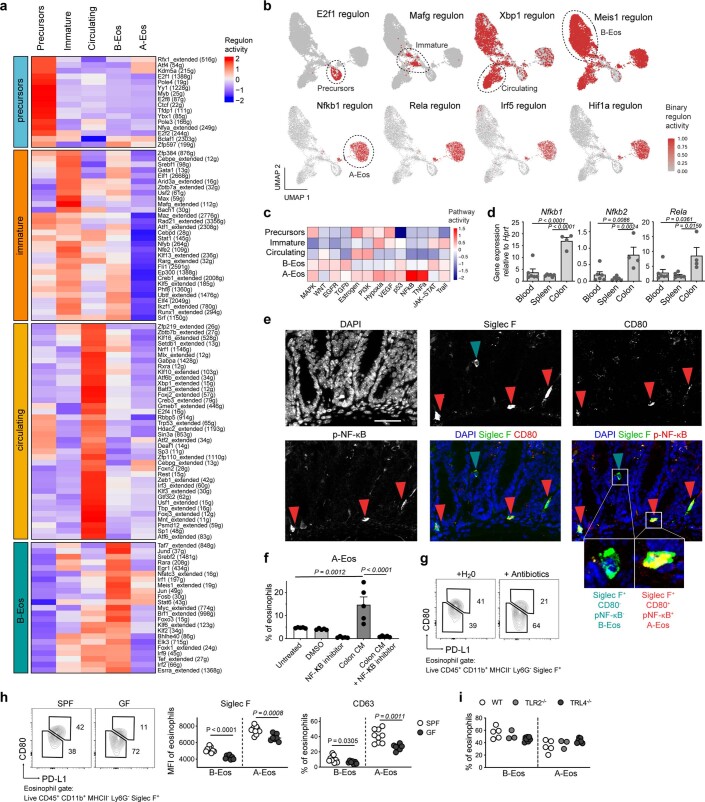
A-Eos are induced by NF-κB signalling. **a**, Regulon activity across clusters. **b**, Representative regulons projected on UMAP plot. Cells are coloured by binary regulon activity. **c**, Pathway activity across clusters according to PROGENy analysis. **d**, Gene expression relative to *Hprt* measured by qRT–PCR of eosinophils sorted from the blood (n = 6), spleen (n = 6) and colon (n = 4) of *Il5*-tg mice. Data represents mean ± SEM. One-way ANOVA. **e**, Representative images of pNF-κB p65 immunofluorescence staining in colonic eosinophils (n = 3, B6J). Arrows mark A-Eos (Siglec-F^+^ CD80^+^, red) and B-Eos (Siglec-F^+^ CD80^−^, green). Nuclei are stained with DAPI. Scale bar, 20 µm. **f**, A-Eos frequencies upon conditioning of BM-Eos with colon CM and/or NF-κB inhibitor (n = 5, B6J). Data represents mean ± SD. One-way ANOVA. **g**, Representative FACS plots of colonic A-Eos (PD-L1^+^CD80^+^) and PD-L1^−^CD80^−^ (B-Eos), relative to Fig 3d. Numbers indicate % of eosinophils. **h**, Left: Representative FACS plots of colonic A-Eos (PD-L1^+^CD80^+^) and PD-L1^−^CD80^−^ (B-Eos), relative to Fig 3e. Numbers indicate % of eosinophils. Right: MFI of Siglec-F and % CD63 in colonic A-Eos and B-Eos shown in Fig. 3e. Medians are shown. Two-tailed unpaired Student’s *t*-test. **i**, Colonic A-Eos and B-Eos frequencies at steady state in B6J (n = 5) Tlr2^−/−^ (n = 3) and Tlr4^−/−^ (n = 7) mice. Medians are shown. One-way ANOVA.

Our data suggest that A-Eos have a dual antibacterial and immunomodulatory role during inflammation. We next sought to acquire a mechanistic understanding of the gene-regulatory network that governs the maturation, function and plasticity of A-Eos. Single-cell regulatory network inference and clustering (SCENIC; ref. ^28^) revealed highly cluster-specific regulon activities and non-overlapping transcription factor profiles (Extended Data Fig. 6a,b). A-Eos exhibited high activity of several NF-κB-related regulons (*Rela*, *Relb*, *Nfkb1* and *Nfkb2*), which were predicted to directly govern the expression of *Cd274* and *Cd80* (Fig. 3a and Extended Data Fig. 6b). In line with the robust activation of this pathway that was indicated by both SCENIC and PROGENy analysis (Extended Data Fig. 6c), NF-κB signalling components were specifically upregulated in A-Eos and were expressed at significantly higher levels in colonic eosinophils compared with their blood and splenic counterparts (Fig 3b and Extended Data Fig. 6d). Furthermore, the co-localization of phosphorylated NF-κB p65 (pNF-κB p65) with CD80^+^, but not CD80^−^, eosinophils in the mouse colonic lamina propria indicates selective activation of canonical NF-κB signalling in A-Eos (Fig 3c and Extended Data Fig. 6e). Notably, NF-κB inhibition in vitro abolished BM-Eos conditioning into A-Eos (Extended Data Fig. 6f).

Owing to their proximity to the lumen, we speculated that A-Eos might be induced by microbiota-derived cues signalling through the TLR–NF-κB pathway. Indeed, the proportion of A-Eos in the colon was significantly reduced after the depletion of commensal bacteria by broad-spectrum antibiotics (Fig. 3d and Extended Data Fig. 6g) as well as in germ-free mice (Fig. 3e and Extended Data Fig. 6h). Germ-free mice also exhibited a marked reduction in eosinophil secretory activity, most prominently in A-Eos (Extended Data Fig. 6h). However, A-Eos frequencies were not affected by TLR2 or TLR4 deficiency (Extended Data Fig. 6i), suggesting independence from these major bacterial recognition pathways.

**Extended Data Fig. 7:**
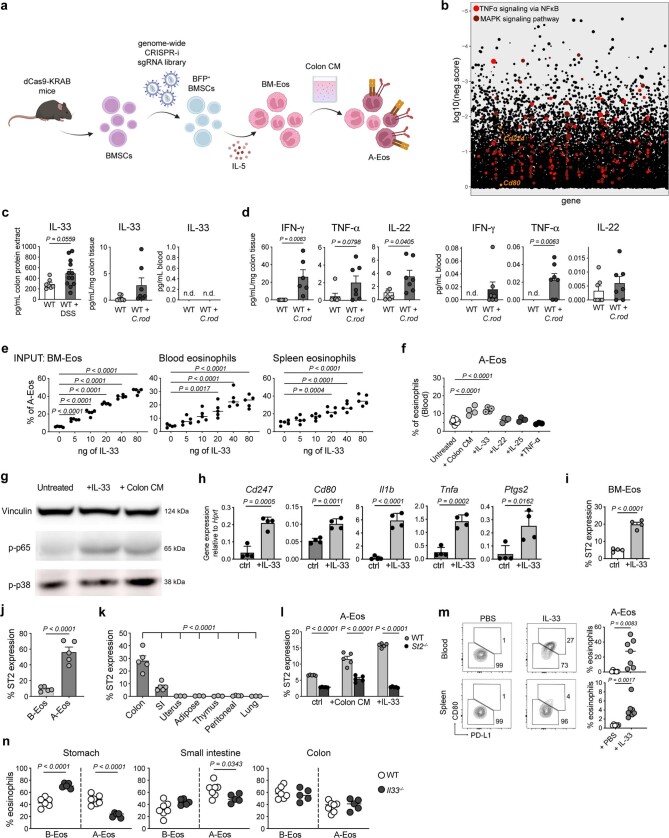
IL-33 induces the accumulation of A-Eos in the colon. **a**, Experimental workflow of the CRISPR inhibition screen. **b**, Log10 negative score per gene, as calculated by MAGeCK. *Cd80* and *Cd274* evidenced in orange. Genes involved in TNF signalling pathway via NF-κB in red, and MAPK signalling pathway in darkred. **c**, IL-33 concentrations measured by ELISA in colon of DSS-treated mice (n = 12, B6J) and colon and blood of *C. rodentium*-infected mice (n = 7, B6J), compared to untreated controls (n = 7, B6J). Medians are shown. Two-tailed unpaired Student’s *t*-test. **d**, IFNγ, TNF and IL-22 concentrations measured by LEGENDplex in colon (left) and blood (right) of *C. rod*entium-infected mice (n = 7, B6J), compared to untreated controls (n = 7, B6J). Medians are shown. Two-tailed unpaired Student’s *t*-test. **e**, A-Eos (PD-L1^+^CD80^+^) frequencies upon conditioning of BM-Eos with colon CM, IL-22, IL-25, TNF or IL-33 (n = 4, B6J). Data are pooled from two independent experiments. Medians are shown. One-way ANOVA. **f**, A-Eos frequencies after conditioning with increasing doses of IL-33. Input: BM-derived (n = 5, B6J), blood (n = 5, *Il5*-tg) and splenic (n = 5, *Il5*-tg) eosinophils. Medians are shown. One-way ANOVA. **g**, Western blot of phospho-p38 and phospho-p65 upon conditioning of BM-Eos with colon CM or IL-33 (n = 3, B6J). **h**, Gene expression normalized to *Hprt* measured by qRT–PCR of BM-Eos upon conditioning with IL-33 (n = 4, B6J). Data represents mean ± SEM. Two-tailed unpaired Student’s *t*-test. **i**, ST2 expression in BM-Eos upon IL-33 treatment (n = 4, B6J). Data represents mean ± SEM. Two-tailed unpaired Student’s *t*-test. **j**, ST2 expression in colonic A-Eos and B-Eos (n = 5, B6J). Data represents mean ± SEM. Two-tailed unpaired Student’s *t*-test. **k**, ST2 expression across organs (n = 5, B6J). Data represents mean ± SEM. One-way ANOVA. **l**, A-Eos frequencies upon conditioning of WT (n = 2, pooled B6J) or ST2^−/−^ (n = 2, pooled) BM-Eos with colon CM or IL-33. Technical replicates and mean ± SEM are shown. Two-tailed unpaired Student’s *t*-test. **m**, Left: Representative FACS plots of A-Eos (PD-L1^+^ CD80^+^) and PD-L1^−^CD80^−^ eosinophils in the blood (top) and spleen (bottom). Numbers indicate % of eosinophils. Right: A-Eos frequencies in mice treated with IL-33 (n = 6-7, B6J). Medians are shown. Two-tailed unpaired Student’s *t*-test. **n**, A-Eos and B-Eos frequencies in the indicated organs of B6J (n = 7) and *Il33*^−/−^ (n = 5) mice at steady state. Medians are shown. Two-tailed unpaired Student’s *t*-test.

To identify regulatory checkpoints of A-Eos differentiation, we conducted an in vitro genome-wide CRISPR inhibition screen (Extended Data Fig. 7a). We found that single guide RNAs (sgRNAs) targeting genes involved in NF-κB and MAPK signalling were significantly depleted in PD-L1^+^CD80^+^ but not in PD-L1^−^CD80^−^ eosinophils, compared to BM stem cells (Fig. 3f and Extended Data Fig. 7b). This observation is in line with our transcriptome analysis (Extended Data Figs. 1f and 6c) and suggests that activation of these pathways is required for A-Eos maturation. Notably, in vitro stimulation with the alarmin IL-33—but not with other cytokines such as IL-22, IL-25 and TNF, the levels of which increase during inflammation (Extended Data Fig. 7c,d)—was sufficient to induce A-Eos marker expression in a dose-dependent manner (Extended Data Fig. 7e,f). Moreover, IL-33 neutralization significantly reduced the differentiation of conditioned BM-Eos into A-Eos (Fig. 3g). Treating BM-Eos with IL-33 quickly led to the phosphorylation of p38 and p65, induced the expression of *Cd274*, *Cd80* and several other A-Eos markers, and further upregulated the surface presentation of the IL-33 receptor ST2 (Extended Data Fig. 7g–i). In vivo, ST2 was expressed at higher levels by A-Eos than B-Eos, suggesting a positive feedback loop to promote tissue adaptation (Extended Data Fig. 7j). Of note, we did not detect ST2 expression in lung, adipose, uterine, peritoneal or thymic eosinophils, which further suggests that the induction of A-Eos by IL-33 may be specific to the GI tract in homeostatic conditions (Extended Data Fig. 7k). IL-33 is known to activate the p38–MAPK and NF-κB pathways via the ST2–MyD88 signalling axis^29^. Indeed, ST2 deficiency abolished the effects of IL-33 treatment in BM-Eos, and significantly reduced their ability to be conditioned by colon CM (Extended Data Fig. 7l). In vivo, treatment with recombinant IL-33 markedly increased the frequencies of A-Eos in the colon and other organs in a MyD88-dependent manner (Fig. 3h and Extended Data Fig. 7m). Finally, A-Eos frequencies at steady state were reduced in the small intestine and stomach of *Il33*^−/^^−^ mice, but not in the colon, indicating that alternative, possibly microbiota-dependent mechanisms may contribute to A-Eos differentiation in the healthy colon (Extended Data Fig. 7n).

## Role of IFNγ in A-Eos regulatory functions in colitis

**Fig. 4:**
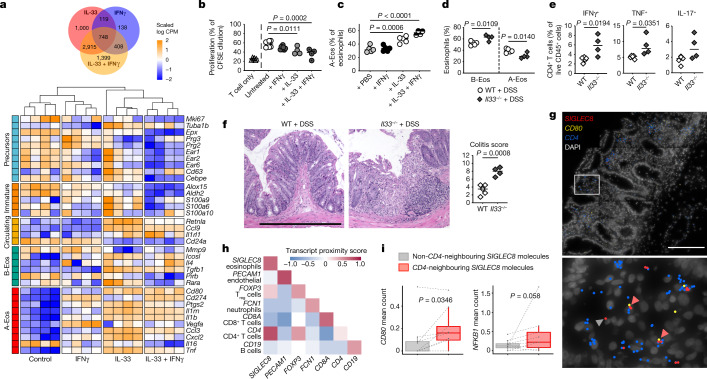
A-Eos co-localize with CD4^+^ T cells in patients with IBD. **a**, Top, Venn diagram of significant differentially expressed genes (DEGs) (false discovery rate (FDR) < 0.05, logFC > |2|) in BM-Eos treated with IL-33 and/or IFNγ (*n* = 4, B6J). All DEGs are listed in Supplementary Table 4. Bottom, expression of subset markers across conditions. Columns are clustered, rows are scaled. CPM, counts per million. **b**, Proliferation of anti-CD3/CD28-activated, CFSE-labelled naive CD4^+^ T cells co-cultured with BM-Eos conditioned with IL-33 and/or IFNγ (*n* = 4, B6J). Data are pooled from two independent experiments. Medians are shown. One-way ANOVA. **c**, A-Eos frequencies in mice treated with IL-33 and/or IFNγ (*n* = 5, B6J). Medians are shown. One-way ANOVA. **d**, A-Eos and B-Eos frequencies in DSS-treated B6J (*n* = 5) and *Il33*^−/^^−^ (n = 4) mice. Medians are shown. Two-tailed unpaired Student’s *t*-test. **e**, Frequencies of IFNγ-, IL-17- and TNF-expressing colonic CD4^+^ T cells from mice in **d**. Medians are shown. Two-tailed unpaired Student’s *t*-test. **f**, Left, representative haematoxylin and eosin (H&E)-stained colonic sections of mice in **d**. Scale bar, 100 µm. Right, colitis score assessed by histopathological examination. Medians are shown. Two-tailed unpaired Student’s *t*-test. **g**, Representative molecular cartography images of human ulcerative colitis samples. Nuclei are stained with DAPI; *CD4*, *SIGLEC8* and *CD80* RNA molecules are shown in blue, red and yellow, respectively. Scale bar, 200 µm. **h**, Pairwise proximity score of transcripts across slides. The score indicates the fraction of slides in which the proximity of a pair of transcripts is significantly higher than expected by chance. *P* values are computed using a permutation test (Methods). T_reg_ cells, regulatory T cells. **i**, Mean counts per slide of *CD80* and *NFKB1* transcripts in the proximity (<10 µm) of *SIGLEC8* transcripts spatially associated with *CD4* molecules versus *SIGLEC8* molecules not associated with *CD4* molecules. The central line in the box plot represents the median count per slide, the lower and upper hinge correspond to the first quartiles and the whisker extends from the hinge to the smallest or largest value no further than 1.5 times the interquartile range (IQR) from the hinge. Two-sided paired Wilcoxon test (17 regions of interest (ROIs), *n* = 4 patients).

**Extended Data Fig. 8:**
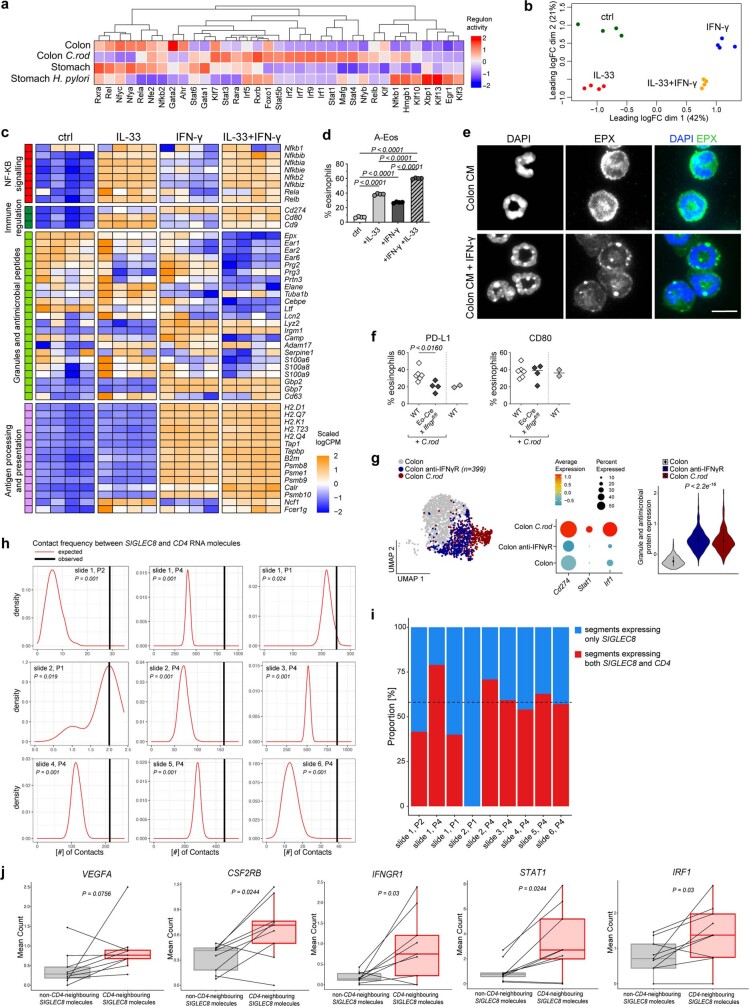
A-Eos co-localize with CD4^+^ T cells in human IBD. **a**, Regulon activity in A-Eos across conditions (n = 4, *Il5*-tg). **b**, Multidimensional scaling (MDS) plot of bulk RNA-seq samples shown in Fig.4a. **c**, Heat map of signature gene expression across conditions of samples shown in Fig. 4a. **d**, A-Eos (PD-L1^+^CD80^+^) frequencies upon treatment of BM-Eos with IL-33 and/or IFNγ. (n = 4, B6J). Data represents mean ± SEM. One-way ANOVA. **e**, EPX immunofluorescence of A-Eos upon exposure to IFNγ for 90 min. Splenic eosinophils were magnetically enriched (n = 2, *Il5*-tg), treated overnight with colon CM and A-Eos sorted by flow cytometry. Scale bar, 10 µm. **f**, Frequencies of PD-L1^+^ and CD80^+^ in colonic eosinophils of WT (n = 6, B6J) and Eo-*Cre;Ifngr*^*fl/fl*^ mice (n = 4) upon *C. rodentium* infection, relative to uninfected controls (n = 2, B6J). Medians are shown. Two-tailed unpaired Student’s *t*-test. **g**, Left: UMAP of single-cell eosinophil transcriptomes isolated from the colon of anti-IFNγR-treated, *C. rodentium*-infected or control *Il5*-tg mice (n = 3). Middle: expression of IFNγ target genes. Right: Expression of granule and antimicrobial signatures. Data represents mean ± SD. Two-sided Wilcoxon test. **h**, Observed vs. expected number of contacts between clusters of *SIGLEC8* and *CD4* molecules shown per slide. P Values are computed based on a two-sided permutation test (see Methods). **i**, Proportions of segmented cells expressing *SIGLEC8* only (blue) or co-expressing both *SIGLEC8* and *CD4* (red) across slides. Dotted horizontal line shows mean. **j**, Mean count per slide of molecules of a given transcript in the proximity (<10 µm) of *SIGLEC8* RNA molecules spatially associated with *CD4* molecules vs *SIGLEC8* molecules not associated with *CD4* molecules. The central line in the box plot represents the median count per slide, the lower and upper hinge corresponds to the first quartiles and the whisker extends from the hinge to the smallest or largest value no further than 1.5 x IQR from the hinge. Two-sided paired Wilcoxon test (17 ROIs, n = 4 patients).

The analysis of our challenge dataset by SCENIC suggests that signalling downstream of IFNγ is increased during inflammation in A-Eos. In particular, *C. rodentium* infection shifted the regulatory landscape towards signalling through STATS (*Stat1*, *Stat3*, *Stat4*, *Stat5b* and *Stat6*) and IRFs (*Irf1*, *Irf2*, *Irf5*, *Irf7* and *Irf9*) (Extended Data Fig. 8a). Notably, *Ifngr1* expression was restricted to the A-Eos subset (Extended Data Fig. 1j), and its deficiency in the eosinophil compartment results in decreased *C. rodentium* clearance and deregulated T cell responses during *H. pylori* infection^23^. To analyse the interplay of IL-33 and IFNγ in regulating A-Eos functions, we performed bulk RNA-seq of BM-Eos treated with IL-33, IFNγ or a combination thereof (Extended Data Fig. 8b). Treatment with IL-33 induced NF-κB signalling and the expression of A-Eos markers, whereas IFNγ treatment strongly upregulated the expression of *Cd274* and genes involved in antigen presentation (Fig. 4a and Extended Data Fig. 8c). Functionally, IL-33 and IFNγ treatment endowed BM-Eos with an increased ability to downregulate the proliferation of CD4^+^ T cells (Fig. 4b). Notably, the synergistic effect of IL-33 and IFNγ not only increased the levels of A-Eos in vitro (Extended Data Fig. 8d), but also shifted the transcriptome of BM-Eos to a more mature state by downregulating granular protein and antimicrobial genes (Fig. 4a and Extended Data Fig. 8c). Treating A-Eos with IFNγ further induced granule mobilization and focal aggregation (Extended Data Fig. 8e). These results suggest a negative feedback loop on the synthesis of granular proteins and antimicrobial peptides, with their release being induced and their transcription being repressed by IFNγ signalling^23,30^.

In vivo, treatment with IFNγ potentiated the effects of IL-33, increasing colonic A-Eos frequencies to the levels observed during colitis (Fig. 4c). Consistently, ablation of IFNGR signalling in the eosinophil compartment impaired the upregulation of PD-L1 in response to infection (Extended Data Fig. 8f). Notably, IFNγR neutralization abrogated *Cd274* expression but did not affect the presence of the A-Eos subset in the steady-state colon, as assessed by scRNA-seq (Extended Data Fig. 8g). In line with our in vitro results, this treatment led to the upregulation of granular protein and antimicrobial peptide genes in A-Eos (Extended Data Fig. 8g).

Our data indicate that IFNγ potentiates but it is not sufficient to induce the A-Eos phenotype, which instead relies on IL-33 signalling. Indeed, IL-33-deficiency prevented the colonic accumulation of A-Eos after treatment with DSS (Fig. 4d). *Il33*^−/^^−^ mice also suffered from increased DSS-induced colitis and did not restrict effector T cell responses (Fig. 4e,f), thus phenocopying eosinophil deficiency. Cumulatively, our data suggest that IL-33 promotes the accumulation of A-Eos during colitis, and that A-Eos limit pathogen incursions and prevent excessive tissue damage through their bactericidal and T-cell-regulatory activities.

## A-Eos co-localize with CD4^+^ T cells in patients with IBD

Finally, we performed multiplexed in situ RNA imaging (molecular cartography) in colon sections from individuals with ulcerative colitis (A.L. et al., manuscript in preparation) and found that *CD4* transcripts significantly co-localized with *SIGLEC8* transcripts (Fig. 4g,h and Extended Data Fig. 8h). Indeed, 55% of *SIGLEC8*^*+*^ segmented areas were also positive for *CD4* (Extended Data Fig. 8i), suggesting that eosinophils and CD4^+^ T cells are in close spatial proximity in the colon of patients with IBD. In a segmentation-free approach, *CD4*-neighbouring *SIGLEC8* RNA molecules were significantly more associated with the A-Eos markers *CD80*, *VEGFA* and *CSF2RB* than were non-*CD4*-neighbouring *SIGLEC8* molecules, indicating that CD4^+^ T cells preferentially interact with A-Eos (Fig. 4i and Extended Data Fig. 8j). *CD4*-neighbouring *SIGLEC8* RNA molecules were also significantly associated with NF-κB (*NFKB1*) and IFNγ (*IFNGR1*, *STAT1* and *IRF1*) signalling components (Fig. 4i and Extended Data Fig. 8j), which indicates that the same pathways might drive interactions between A-Eos and CD4^+^ T cells in mouse and human colitis.

## Discussion

Neutralizing antibodies against the cytokine IL-5 are widely used in severe eosinophilic asthma to inhibit the differentiation of eosinophils^31^. Whether intestinal eosinophils can be exploited as therapeutic targets in IBD is still unknown, and thus a thorough investigation of their functions in the healthy and inflamed gut is warranted. Owing to the technical challenges involved in profiling these elusive cells, eosinophils have long remained overlooked in colitis. Here, we identify a subset of GI-resident eosinophils that are enriched in patients with IBD and in experimental models of colitis. In response to bacterial cues, IL-33 and IFNγ signalling, A-Eos exert a protective role on the intestinal mucosa by means of their antibacterial and immunomodulatory activity. Impaired accumulation of A-Eos in the inflamed colon worsens inflammation and leads to the hyperactivation of CD4^+^ T cells during acute colitis. However, the strong effector and cytotoxic potential of this subset can potentially also favour tissue damage in settings of chronic inflammation. More studies are needed to elucidate the extent and nature of their protective activities in human intestinal homeostasis and inflammation, and whether these can be targeted for the treatment of IBD.

## Methods

### Mice

All experiments were performed on 6–16-week-old mice. Mice of a given strain were randomly divided into the different groups and both males and females were included in studies. Treatments and study termination was performed by two or more experimenters and blinding during result assessment was done by converting animal identifiers into numbers during sample processing and analysis. C57BL/6J (B6J, stock no. 000664) and dCas9-KRAB (stock no.030000) mice were obtained from The Jackson Laboratory; OT-1 (stock no. 003831), OT-II (stock no. 004194), *MyD88*^−/^^−^ (ref. ^32^), *Tlr2*^−/^^−^ (stock no. 004650), CD45.1 (stock no. 002014) and *Tlr4*^−/^^−^ (ref. ^33^) mice were obtained from a local live mouse repository. *Id2*^*CreERT2*^*;Rosa26*^*EYFP*^ mice^34^, *Il5*-transgenic mice^35^ and *Ifngr2*^*fl/fl*^ mice^36^ have been previously described. *Il33*^−/^^−^ (ref. ^37^) were obtained through the RIKEN Center for Developmental Biology (accession number CDB0631K) and *St2*^−/^^−^(ref. ^38^) mice have been described and were backcrossed onto a C57BL/6J background. Eosinophil-deficient mice^39^ (PHIL) and mice expressing Cre under the EPX promoter^40^ (Eo-*Cre*) were obtained from J. J. Lee. Chow and water were available ad libitum, unless specified. All mice were in the B6J background and maintained on a 12-h light–12-h darkness schedule. Mice were housed and bred under specific-pathogen-free conditions in accredited animal facilities. Germ-free mice were bred and maintained in open-top cages within flexible-film isolators, supplied with HEPA-filtered air, and autoclaved food and water ad libitum. At the experimental end-point, mice were euthanized by increasing CO_2_ concentrations. All experimental procedures at the University of Zurich and Bern were performed in accordance with Swiss Federal regulations and approved by the Cantonal Veterinary Office and/or in accordance with the European Communities Council Directive (86/609/EEC), Czech national guidelines, institutional guidelines of the Institute of Molecular Genetics and approved by the Animal Care Committee.

### Animal experiments

#### Antibody neutralization

For the 10-day treatment: 7–8-week-old female and male mice (B6J) were injected intraperitoneally twice a week with 0.5 mg anti-IL-5 (BE0198 BioXCell, TREK5) or anti-keyhole limpet haemocyanin isotype control (BE0090, BioXCell, LTF-2), or anti-IFNγR (BE0029, BioXCell, GR-20) or anti-CCR3 (BE0316 clone 6S2-19-49) or anti-horseradish peroxidase isotype control (BE0088, BioXCell, HRPN) antibodies for 10 days before the study end-point.

#### Depletion of intestinal commensals by antibiotic treatment

Seven-to-eight-week-old female mice (B6J) were treated for 10 consecutive days with ampicillin (1 g l^−1^; A0166 Sigma), vancomycin (500 mg l^−1^; A1839,0001 Applichem), neomycin sulfate (1 g l^−1^; 4801 Applichem), and metronidazole (1 g l^−1^; H60258 Alfa Aesar) in autoclaved drinking water, as previously described^41^. Water bottles were monitored and refilled twice per week.

#### Adoptive transfer

A total of 10^6^ magnetically selected splenic eosinophils of 6–12-week-old *Il5*-tg female and male mice were injected intravenously in 100 µl PBS into CD45.1 recipients (8–12-week-old female and male mice). Organs were collected 42 h after injection.

#### DSS-induced colitis

Six-to-twelve-week-old female and male mice (PHIL, B6J and *Il33*^−/−^) were treated with 2.5% DSS (w/v; 9011-18-1, MP Biomedicals) dissolved in autoclaved drinking water for five days, followed by three days of regular water, before organs were collected. Water bottles were monitored and refilled twice per week.

#### *H. pylori* challenge

Six-to-twelve-week-old female and male mice (*Il5*-tg and B6J) were infected orally with the *H. pylori* strain PMSS1 (10^7^ colony-forming units, CFU) and analysed four weeks after infection. The PMSS1 strain, a clinical isolate of a patient with a duodenal ulcer, was grown on horse blood agar plates followed by liquid culture, as previously described^23^. Cultures were routinely assessed by light microscopy for contamination, morphology and motility. *C. rodentium:* 6–12-week-old female and male mice (*Il5*-tg and B6J) were infected orally with the nalidixic-acid-resistant *C. rodentium* strain ICC169 (ATCC 51549, 10^8^ CFU) and analysed 13 days after infection. Bioluminescent *C. rodentium* strains ICC180 (ICC169 derivative, nalidixic-acid- and kanamycin-resistant) was a gift from G. M. Frankel and was previously described^42^. Both strains were grown on agar plates (1.5%; A0927 Applichem), followed by single-colony picking and overnight culture in antibiotic-supplemented Luria broth (nalidixic acid, 50 μg ml^−1^; N4382 Sigma and/or kanamycin, 50 μg ml^−1^; 420311 Sigma).

#### Cytokine administration

Seven-to-eight-week-old female mice (*MyD88*^−/−^ and B6J) were injected intraperitoneally every other day with three total doses of 0.5 mg rec-IL-33 (210-33, PeproTech) and/or IFNγ (315-05, PeproTech) or with PBS control.

#### EdU labelling

Seven-to-eight-week-old female and male mice (*Il5*-tg and B6J) were infected orally with *C. rodentium* or left uninfected; four days before analysis mice were injected with EdU (2.5 mg per mouse, 900584 Sigma).

#### HDM challenge

Four-month-old female mice (B6J) received 1 µg HDM extract in 50 µl PBS intratracheally for sensitization (day 0) and were then challenged once a day with 10 µg HDM in 50 µl PBS for 5 days (day 7–11). Lungs were collected 14 days after the sensitization.

#### Tamoxifen injection

Six-to-twelve-week-old female and male mice (*Id2*^*CreERT2*^*;Rosa26*^*EYFP*^) were gavaged with a single dose of tamoxifen (T5648 Sigma). Tamoxifen was dissolved in a small volume of 100% ethanol (pre-warmed at 50 °C) and then resuspended in corn oil (pre-warmed at 50 °C) to the final concentration of 5 mg per mouse. Organs were collected 2 h, 2 and 4 days after the injection.

### Preparation of single-cell suspensions from tissues

#### GI tissues

Stomach, colon and small intestine were collected, cleaned of faecal matter and cut longitudinally. Organs were washed in PSB and cut into pieces (1–2 cm) and Peyer’s patches were removed from the small intestine. Pieces were washed twice in a shaking incubator with wash buffer (2% BSA, 100 U ml^−1^ penicillin–streptomycin and 5 mM EDTA in HBSS, 25 min, 37 °C). Tissues were then rinsed in cold PBS and digested for 50 min at 37 °C in complete medium (10% FBS and 100 U ml^−1^ penicillin–streptomycin (P0781 Sigma) in RPMI-1640) containing 15 mM HEPES (H0887 Sigma), 0.05 mg ml^−1^ DNase I (10104159001 Roche) and an equal amount of 250 U ml^−1^ type IV (C5138 Sigma) and type VIII collagenase (C2139 Sigma) (for colon and small intestine), or 500 U ml^−1^ type IV collagenase (C5138 Sigma) (for stomach). Cells were passed through a 70-μm cell strainer, centrifuged for 8 min and layered onto a 40/80% Percoll (17089101 Cytiva) gradient (18 min, 2,100*g*, 20 °C, no brake). The interphase was collected and washed in PBS.

#### Lung

Lungs were perfused with PBS, collected and cut into pieces before digestion in complete medium supplemented with 500 U ml^−1^ type IV collagenase (Sigma) and 0.05 mg ml^−1^ DNase I (Roche) for 50 min at 37 °C. Lungs were then passed through a 70-μm cell strainer and mesh with syringe plungers. To reduce macrophage contamination (Siglec-F^+^), cells were plated in complete RPMI medium for 1 h at 37 °C.

#### Blood

Blood was sampled by post mortem cardiac puncture in 2% BSA 5 mM EDTA PBS. For *Il5*-tg mice, the suspension was layered over Histopaque 1119 (density of 1.119 g ml^−1^; 11191 Sigma-Aldrich) and centrifuged at 800*g* for 20 min and the interphase was washed in PBS. Red blood cells were lysed in ice-cold distilled water for 30 s.

#### Bone marrow

Femur and tibia were flushed using complete RPMI medium and a 23-gauge needle. The content was collected, filtered through a 40-μm cell strainer and red blood cells were lysed in ice-cold distilled water for 30 s.

#### Spleen, lymph nodes and thymus

Spleen and lymph nodes were collected and meshed through a 40-μm cell strainer using a syringe plunger, and red blood cells were lysed in ice-cold distilled water for 30 s.

#### Peritoneal fluid

Peritoneal cavity was perfused with 5 ml PBS with a 21-gauge needle and the inflated area was massaged for 30 s, to disperse the solution. The peritoneal liquid was collected and cells were plated in complete RPMI medium for 1 h at 37 °C to remove adherent cells.

#### Adipose tissue

Lungs were perfused with PBS and the perigonadal adipose depot was isolated, removing any visible gonadal tissue. The tissue was minced into small pieces and digested in complete DMEM medium supplemented with 0.2 mg ml^−1^ Liberase (05401020001 Roche) and 0.05 mg ml^−1^ DNase I (Roche) for 50 min at 37 °C. Suspensions were filtered through a 100-μm cell strainer and centrifuged at 1,000*g* for 10 min. The pellet was collected and washed in PBS.

#### Uterus

Uterus was collected, cut longitudinally and washed in PBS. Pieces were shaken in wash buffer (2% BSA, 100 U ml^−1^ penicillin–streptomycin and 5 mM EDTA in HBSS, 25 min, 37 °C). The tissue was then rinsed in cold PBS and digested for 50 min at 37 °C in complete medium containing 0.05 mg ml^−1^ DNase I (Roche) and 0.2 mg ml^−1^ Liberase (Roche). Cells were passed through a 70-μm cell strainer, centrifuged and washed in PBS.

Unless specified, all centrifugation steps were performed at 500*g* for 8 min at 10 °C.

### Magnetic cell enrichment

Eosinophils of 6–12-week-old female and male mice (*Il5*-tg) were positively enriched using a PE anti-mouse Siglec-F antibody (562068 BD Biosciences; E50-2440) and anti-PE microbeads (130-042-401 Miltenyi Biotech), according to the manufacturer’s instructions. Immune cells of 7–8-week-old female mice (B6J) were positively enriched using anti-CD45 microbeads (130-052-301 Miltenyi Biotech), according to the manufacturer’s instructions.

### scRNA-seq

#### Single-cell capture and library preparation

Whole-transcriptome analyses of magnetically enriched Siglec-F^+^ eosinophils (blood, spleen, stomach, colon, small intestine, adipose tissue, lung and uterus, *Il5-*tg), total BM cells (*Il5-*tg) or CD4^+^ cells (colon, B6J) were performed using the BD Rhapsody Single-Cell Analysis System (BD, Biosciences). Cells were pooled from three to five mice per sample. Tissue processing and enrichment procedures are described above. Each preparation was assessed by flow cytometry to determine eosinophil viability and was subjected to morphological examination after cytospin and staining. Eosinophils were labelled with sample tags (633793 BD Mouse Single-Cell Multiplexing Kit) according to the manufacturer’s protocol. In brief, for each condition, 10^6^ cells were resuspended in staining buffer (1% BSA, 1% EDTA in PBS) and incubated with the respective Sample Tag for 20 min at room temperature. Cells were then transferred to a 5-ml polystyrene tube, washed twice with 2 ml staining buffer and centrifuged at 400*g* for 5 min. Samples were resuspended in 1 ml staining buffer for counting. Next, 10,000 or 20,000 cells from up to 4 barcoded samples were pooled for a total of 60,000 cells and the mixture was centrifuged at 400*g* for 5 min. The pellet was resuspended in 650 BD Sample Buffer supplemented with 1:1,000 SUPERase in (20 U µl^−1^; AM2694 Thermo Fisher Scientific) and NxGen Rnase Inhibitor (40 U µl^−1^; 30281-2 Lucigen). BD Rhapsody cartridges were super-loaded with 60,000 cells each. Single cells were isolated with the BD Rhapsody Express Single-Cell Analysis System according to the manufacturer’s recommendations (BD Biosciences). cDNA libraries were prepared using the BD Rhapsody Whole Transcriptome Analysis Amplification Kit (633801 BD Biosciences) following the BD Rhapsody System mRNA Whole Transcriptome Analysis (WTA) and Sample Tag Library Preparation Protocol (BD Biosciences). The final libraries were quantified using a Qubit Fluorometer with the Qubit dsDNA HS Kit (Q32851 Thermo Fisher Scientific). Library size distribution was measured with the Agilent high-sensitivity D5000 assay on a TapeStation 4200 system (5067-5592 Agilent Technologies). Sequencing was performed in paired-end mode (2 × 75 cycles) on a NovaSeq 6000 with NovaSeq 6000 SP Reagent Kit chemistry.

#### Data pre-processing and normalization

After demultiplexing of bcl files with Bcl2fastq v.2.20.0.422 (Illumina) and quality control, paired-end scRNA-seq FASTQ files were processed on the Seven Bridges Genomics platform with default parameters. Downstream analysis was conducted in R v.4.1.0 with the package Seurat v.4.0.3 (ref. ^43^). All Seurat objects (one for each of the multiplexed samples) were merged and subjected to the same quality filtering. Cells with fewer than 200 or more than 2,500 detected genes were excluded from the analysis. After log normalization, the count data were scaled regressing for mitochondrial reads, and principal component analysis (PCA) was performed based on the 2,000 most variable features. Clustering and UMAP visualization were performed on the merged dataset using 50 principal components and a resolution of 0.3 for the shared nearest neighbour clustering algorithm. The clusters were annotated manually on the basis of marker gene expression. Epithelial and mesenchymal contaminants, as well as immune-cell clusters not belonging to the eosinophil lineage, were excluded from downstream analysis. A cluster high in mitochondrial genes was excluded as well. The eosinophil space was analysed by subsetting clusters expressing eosinophil markers. The subsetted dataset was subjected to normalization, scaling and PCA as above. Clustering and UMAP visualization was performed using 20 principal components and a resolution of 0.3 for the shared nearest neighbour clustering algorithm. For the lung, uterus and adipose tissue dataset, batch correction was performed with Harmony^44^ and epithelial genes (marker genes of epithelial cluster with pct.2 < 0.05) derived from excessive-cell-free RNA were removed from the counts.

#### Differential gene expression analysis, gene-set enrichment and score computation

To extract cluster markers, FindAllMarkers was executed with logfc.threshold and min.pct cut-offs set to 0.25. Top-ranked genes (by log fold change; logFC) were extracted for illustration. For differential gene expression, FindMarkers was applied with logfc.threshold and min.pct set to 0. Genes were subsequently filtered on the basis of Bonferroni-adjusted *P* < 0.05. Scores were computed with the AddModuleScore function. Genes used for the scores and signatures were manually curated from Gene Ontology (GO) terms and literature, and are listed in Supplementary Table 3. Cell-cycle scoring was performed with the CellCycleScoring algorithm from Seurat, using cell-cycle-related genes^45^. For gene-set enrichment analysis (GSEA), differentially expressed genes were pre-ranked in decreasing order by the negative logarithm of their *P* value, multiplied for the sign of their average logFC (in R, ‘- log(p_val)*sign(avg_log2FC)’). GSEA was performed on this pre-ranked list using the R package FGSEA (https://github.com/ctlab/fgsea/) with default parameters and the GO Biological Process database, made accessible in R by the package msigdbr (https://github.com/cran/msigdbr). The results were filtered for significantly enriched gene sets (Bonferroni-adjusted *P* < 0.05).

#### Trajectory inference and trajectory alignment

Trajectory inference was performed with Monocle 2.3.6 (refs. ^19,46^) in R v.3.6.3. After creating a Monocle object using ‘negbinomial.size()’ distribution and lowerDetectionLimit = 0.5, the analysis was performed using Seurat’s top 2,000 variable features as ordering genes. Dimensionality reduction was performed using the DDTree method. To visualize the eosinophil differentiation, cluster annotations were projected on the inferred trajectories. Trajectory alignment of the BM–blood–colon trajectories was performed by applying dynamic time warping as described previously^22,47^. The steady-state and *C. rodentium*-challenge trajectories were set as the reference and query, respectively. Differentially expressed genes were identifying by using a full model of ‘y ~ pseudotime*treatment’ and a reduced model of ‘y ~ pseudotime’.

#### RNA velocity and cell fate probabilities

Loom files were generated with velocyto^48^ and dynamical velocities were computed with scvelo^20^. Fate probabilities were computed with CellRank^21^ and plotted as pie charts (partition-based graph abstraction, PAGA).

#### Analysis of pathway and regulon activity

Pathway activity was calculated across eosinophil subsets with PROGENy v.1.13.2 (ref. ^49^) with default parameters. Gene-regulatory activity was interrogated by applying SCENIC 1.2.4 (ref. ^28^) with default parameters. In brief, after expression matrix filtering (minCountsPerGene = 3*.01*ncol(exprMat), minSamples = ncol(exprMat)*.01), and computing correlation, GENIE3 was applied to infer potential transcription factor targets. Co-expression networks were then calculated, regulons were created and their activity was scored in cells. Regulon activities were visualized as cluster averages using the R package ComplexHeatmap (ref. ^50^).

#### Integration of datasets

Challenge, DSS and B6J datasets were integrated using Seurat’s anchoring-based integration method using the steady-state object as reference dataset (reference.reduction = “pca”, dims = 1:50).

#### Prediction of cell–cell interaction with CellPhoneDB

Ligand–receptor interaction analysis was performed using the Python package CellPhoneDB (v.2.0.0, Python v.3.8.5) following instructions from the GitHub repository (https://github.com/Teichlab/cellphonedb). In brief, the annotated Seurat object of isolated lamina propria immune cells from DSS-treated B6J mice was used to test the expression of known ligand–receptor interactions from the public repository of CellPhoneDB. Gene symbols were first converted from mouse to human using the biomart R package (v.2.46.3). Mean values representing the average ligand and receptor expression of annotated clusters were calculated on the basis of the percentage of cells expressing the gene, and the gene-expression mean. To determine the significance of observed means, *P* values were calculated using a null distribution of means calculated for randomly permuted annotated cluster labels. An interaction was considered significant if *P* ≤ 0.05. Significant ligand–receptor interaction pairs between eosinophils and CD8^+^ T cells or CD4^+^ T cells were extracted, gene symbols were converted from human to mouse and their mean values were plotted using the plot_cpdb function from the ktplots R package (v.1.1.14) (https://github.com/zktuong/ktplots).

#### Plotting and statistical analysis

Statistical analysis and visualization were performed using R version 3.6.3 or 4.1.0. Statistical significance tests were performed as described in each figure legend. Unless stated otherwise, all tests were significant with Bonferroni-adjusted *P* < 0.05. Plots were generated with the R package ggplot2 (ref. ^51^).

### Flow cytometry, cell sorting and counting

#### Staining

For surface staining, cells were stained in PBS at 4 °C for 30 min with the fixable viability dye eFluor 780 (1:1,000, 65-0865-14 eBioscience) and a combination of the following antibodies (1:200, all from BioLegend; unless stated otherwise): anti-mouse CD45 BV650 (30-F11, 103151), CD11b BV510 (M1/70, 101263), MHC-II AF700 (M5/114.15.2, 107622), Ly6G Percp-Cy5.5 (1A8, 127616), CD4 PerCP (RM4-5, 100538), TCRβ PE-Cy7 (H57-597, 109222), TCRβ PE-Cy7 (H57-597, 109228), CD80 BV605 (1:100, 16-10A1, 104729), PD-L1 PE-Cy7 (1:100, 10F.9G2, 124314), CD31 PE (390, 102408), CD45.2 BV785 (1:50, 104, 109839), CD9 PE (MZ3, 124805), CD54 BV711 (YN1/1.7.4, 116143), CD63 PE (1:100, NVG-2, 143904), CD95 PE-Cy7(SA367H8, 152607), Siglec-E PE (M1304A01, 677104), SCA-1 AF488 (D7, 108116), SCA-1 AF700 (D7, 108142), C-kit BV605 (ACK2, 135121), CD11c APC-Cy7 (N418, 117323), CLEC12a PE (5D3, 143404), CD49d FITC (R1-2, 103605), CD16/32 FITC (S17012B, 101305), CD3e Percp-Cy5.5 (145-2C11, 100328), CD8a APC (53-6.7, 100712), NK1.1 Percp-Cy5.5 (PK136, 108727), B220 Percp-Cy5.5 (RA3-6B2, 103236), Ter119 Percp (TER-119, 116227), Gr1 Percp (RB6-8C5, 108427), CD34 AF647 (RAM34, 560230), Siglec-F BV421 (E50-2440, 552681 BD Biosciences), Siglec-F PE (E50-2440, 552126 BD Biosciences), CD125 PE (T21, 558488 BD Biosciences), CD275 (HK5.3, 50598582 eBioscience) and T1/ST2 FITC (1:100, DJ8, 101001F MD Bioproductos GmbH). For T cell intracellular cytokine staining, cells were incubated for 3 h 15 min in complete IMDM medium containing 0.1 μM phorbol 12-myristate 13-acetate (P-8139 Sigma) and 1 μM ionomycin (I-0634 Sigma) with 1:1,000 Brefeldin A (00-4506-51 eBioscience) and GolgiStop solutions (51-2092KZ BD Biosciences) in a humidified incubator with 5% CO_2_ at 37 °C. After surface staining, cells were fixed and permeabilized with the Cytofix/Cytoperm Fixation/Permeabilization Solution kit (512090KZ BD Biosciences) according to the manufacturer’s instructions. Cells were then stained for 50 min with anti-mouse IL-17A APC (TC11-18H10.1, 506916), IFNγ BV421 (XMG1.2, 505830) and TNF FITC (MP6-XT22, 506 304) all from Biolegend at 1:100. Fc block (anti-CD16/CD32, 101302 Affymetrix) was included to minimize nonspecific antibody binding. Total leukocyte counts were determined by adding countBright Absolute Counting Beads (C36950 Life Technologies) to each sample before analysis. Samples were acquired in a LSRII Fortessa or FACS AriaIII 5L (BD Biosciences). For high-dimensional spectral flow cytometry analysis, cells were acquired on Cytek Aurora 5L (Cytek Biosciences) following 50 min staining at 4 °C with the antibodies described in Supplementary Table 5. For the Click-iT Plus EdU Alexa Fluor 647 Flow Cytometry Assay Kit (C10419 Thermo Fisher Scientific), the staining protocol was followed according to the manufacturer’s instructions. BD FACSDiva Software (BD Biosciences) was used for data acquisition and cell sorting.

#### Data analysis and plotting

Flow cytometry data analysis was performed with FlowJo software (v.10.7.1 Becton Dickinson). Cell counts, relative cell frequencies or MFI were used to generate graphical plots in GraphPad Prism (v.9.1.1, GraphPad). High-dimensional flow cytometry data were compensated and exported with FlowJo software (v.10) and the resulting FCS files were uploaded into Rstudio (v.4.0.3 R software environment). UMAPs were generated on stochastically selected cells from each sample and FlowSOM metaclusterings were performed for all the exported events as described previously^52^.

#### Statistical analysis

All statistical analyses were performed with GraphPad Prism (v.9.1.1, GraphPad). Two-tailed unpaired Student’s *t*-tests were used for comparing two groups, and comparisons of more than two datasets were done using a one-way analysis of variance (ANOVA) with Tukey’s post-test. Differences were considered statistically significant when *P* < 0.05.

### Isolation and culture of mouse BM-derived eosinophils

To generate mouse BM-derived eosinophils (BM-Eos), BM cell suspensions were seeded at a density of 10^6^ cells per ml in RPMI-1640 medium supplemented with 20% heat-inactivated FBS, 25 mM HEPES (H0887 Sigma), 100 U ml^−1^ penicillin–streptomycin (P0781 Sigma), 2 mM glutamine (25030-024 Gibco), 1× NEAA (11140-035 Gibco), and 1 mM sodium pyruvate (11360070 Gibco). Cells were cultured in a humidified incubator with 5% CO_2,_ 37 °C, and were supplemented with 100 ng ml^−1^ mouse SCF (250-03 PeproTech) and 100 ng ml^−1^ mouse FLT3-Ligand (250-31L PeproTech) from day 0 to day 4, followed by differentiation with 10 ng ml^−1^ mouse rec-IL-5 (215-15 PeproTech) until day 13, as described^53^. Half of the medium was replaced and the cell concentration was adjusted to 10^6^ cells per ml every other day. On day 8, cells were collected and moved to new flasks to remove adherent contaminating cells. On day 13, the nonadherent cells were collected and washed with PBS. Eosinophils were sorted and purity was assessed by flow cytometry (higher than 95%).

### In vitro conditioning with supernatant of cultured colonic explants and cytokines

Supernatant of cultured colonic explants (colon CM) was prepared by culturing mid-colon sections (around 0.3 cm) from 6–12-week-old female and male mice (B6J) in 300 µl complete RPMI medium in a humidified incubator with 5% CO_2_, 24 h at 37 °C. Flow-cytometry-purified eosinophils were magnetically isolated from blood and spleen (*Il5*-tg) or differentiated from the BM (B6J) and were kept in complete RPMI medium with recombinant mouse IL-5 (10 ng ml^−1^, PeproTech). Cells were seeded in round-bottom 96-well plates at a density of 2 × 10^5^ cells per well (100 µl) and conditioned for 12 h at 37 °C with cell-free colon CM (1:10 or at the indicated doses) or the following cytokines: IL-22 (10 ng ml^−1^, 210-22 PeproTech), IL-25 (10 ng ml^−1^, 210-17E PeproTech), TNF (10 ng ml^−1^, 315-01A PeproTech) and IL-33 (20 ng ml^−1^ or at the indicated doses, PeproTech). The NF-κB inhibitor BAY11-7082 (B5556, Sigma) was added at a concentration of 5 μM and anti-IL-33 neutralizing antibody (AF3626, Biotechne) at 30 ng ml^−1^. To study granule mobilization, magnetically enriched splenic eosinophils (*Il5*-tg) were treated overnight with colon CM (1:10) and flow-cytometry-sorted A-Eos were conditioned with IFNγ (20 ng ml^−1^, PeproTech) for 90 min.

### *C. rodentium* ICC180 viability assay

Flow-cytometry-purified BM-Eos (B6J) or magnetically enriched colonic, splenic and blood eosinophils (*Il5*-tg) from 6–12-week-old female and male mice were used for the assay. BM-Eos were conditioned overnight with colon CM (1:10) at 37 °C. Eosinophils were washed with PBS and transferred to a white flat-bottom 96-well plate (Corning) in antibiotic-free RPMI-1640 medium supplemented with 10% FBS and mouse IL-5 (10 ng ml^−1^, PeproTech). A total of 10^8^ bioluminescent *C. rodentium* bacteria (at exponential phase, optical density at 600 nm (OD_600 nm_) = 1–1.5) was added to each well and luminescence was measured after 60 min on an Infinite 200 PRO plate reader (TECAN).

### T cell proliferation assay

Flow-cytometry-purified BM-Eos (B6J) or magnetically enriched splenic eosinophils (*Il5*-tg) or A-Eos and B-Eos sorted from the GI tract (*Il5*-tg) were isolated from 6–12-week-old female and male mice. BM-Eos or spleen-derived eosinophils were conditioned overnight with colon CM (1:10) or treated with recombinant mouse IFNγ (10 ng ml^−1^, PeproTech) and/or IL-33 (20 ng ml^−1^, PeproTech), as indicated. Naive CD4^+^ T cells were isolated from the lymph nodes of 6–12-week-old female and male mice (B6J), enriched with the MojoSort Mouse CD4 Naïve T Cell Isolation Kit (480040 BioLegend) and purified by flow cytometry. T cells were labelled with the CellTrace CFSE Cell Proliferation Kit (C34554 Thermo Fisher Scientific) following the manufacturer’s instructions. T cells were then activated by CD3/CD28 T-activator Dynabeads (11131D Gibco) and co-cultured with eosinophils at a 1:1 ratio (2 × 10^5^ total) for 4 days at 37 °C in complete RPMI medium supplemented with 10 ng ml^−1^ recombinant mouse IL-5 (PeproTech) and 20 ng ml^−1^ IL-2 (402-ML R&D). CFSE dilution was assessed by flow cytometry.

### Antigen presentation assay

BM-Eos were isolated from 6–8-week-old female and male mice (B6J) and purified by flow cytometry. Eosinophils were conditioned overnight with colon CM, where indicated. Cells were washed in PBS and loaded with 300 n ml^−1^ of ovalbumin (OVA) residues 257–264 (S7951 Sigma) or 323–339 (O1641 Sigma) for 6 h in complete RPMI medium supplemented with 10 ng ml^−1^ recombinant IL-5 (PeproTech). T cells were sorted by flow cytometry and labelled with CellTrace CFSE Cell Proliferation Kit (C34554 Thermo Fisher Scientific) following the manufacturer’s instructions. OT-I CD8^+^ and OT-II CD4^+^ T cells were obtained from the lymph nodes of 8–12-week-old female and male mice (OT-I and OT-II, respectively). T cells were co-cultured with eosinophils at a 1:1 ratio (2 × 10^5^ total) for 4 days at 37 °C in complete RPMI medium supplemented with 10 ng ml^−1^ recombinant mouse IL-5 (PeproTech) and 20 ng ml^−1^ IL-2 (402-ML R&D). CFSE dilution was assessed by flow cytometry.

### Quantitative PCR with reverse transcription (qRT–PCR)

The RNA from cultured BM-Eos (B6J) or A-Eos and B-Eos sorted from the small intestine (*Il5*-tg) was isolated using the Direct-zol RNA MicroPrep kit (R2062 Zymo Research), whereas the RNA from magnetically enriched colonic, splenic and blood eosinophils from 6–12-week-old female and male mice (*Il5*-tg) was isolated using the RNeasy Mini kit (74106 QIAGEN). Both isolations were performed according to the manufacturer’s instructions, including the on-column DNase 1 digestion step. Complementary DNA synthesis was performed using Superscript III reverse transcription (18080-044 QIAGEN). Gene expression was measured on a CFX384 Touch Real-Time PCR system (Bio-Rad, Second Derivative Maximum method analysis with high-confidence algorithm) by TaqMan Gene Expression Assays (4331182 Applied Biosystems by Thermo Fisher Scientific): *Cxcl2* (Mm00436450_m1), *Hprt* (Mm03024075_m1), *Gapdh* (Mm99999915_g1), *Cd274* (Mm03048248_m1), *Cd80* (Mm00711660_m1), *Ahr* (Mm00478932_m1), *Nfkb1* (Mm00476361_m1), *Nfkb2* (Mm00479807_m1), *Rela* (Mm00501346_m1), *Tnfa* (Mm00443258_m1), *Il1b* (Mm00434228_m1) and *Ptgs2* (Mm00478374_m1). Gene-expression levels for each sample were normalized to *Hprt* or *Gapdh* expression. Mean relative gene expression was determined, and the differences calculated using the 2ΔC(t) method.

### Bulk RNA sequencing

BM-Eos were isolated from seven-to-eight-week-old female and male mice (B6J), differentiated and purified by flow cytometry. Cells were plated at a density of 5 × 10^5^ cells per well (250 µl) and conditioned overnight with recombinant IL-33 (20 ng ml^−^^1^ PeproTech) and/or IFNγ (15 ng ml^−^^1^ PeproTech). RNA isolation was performed with the RNeasy Mini kit (74106 QIAGEN) according to the manufacturer’s instructions, including the on-column DNase 1 digestion step. RNA quality was assessed by Tapestation (Agilent). Library preparation was performed with the Illumina TruSeq RNA Kit. RNA sequencing was performed on the Illumina Novaseq 6000 (200 Mio reads), single-end read 100 bp. Reads were quality-checked with FastQC. Read alignment to the reference genome Mus_musculus.GRCm39 and read count was performed on the Support Users for SHell script Integration (SUSHI) framework^54^, with the RSEMApp application. Filtering and differential expression testing were performed with edgeR (ref. ^55^). The package pheatmap (ref. ^56^) was used to generate heat maps.

### Immunofluorescence

#### Mouse colonic sections

The colon of 7–8-week-old female and male mice (B6J) was dissected out, flushed in PBS and fixed 3 h in PFA (4% in PBS) at 4 °C, followed by overnight incubation in sucrose (30% w/v in 4% PFA) at 4 °C. Tissue was embedded in Tissue-Tek OCT Compound (Sakura, 4583) and stored at −80 °C. Tissue from three or four mice was cryosectioned (8 µm) onto the same microscope slide, washed in PBS and incubated for 1 h in blocking solution (2.5% BSA, 5% heat-inactivated normal goat serum, 0.1% Tween-20 in PBS) at room temperature. Slides were incubated overnight in blocking solution with the following primary antibodies (1:100): rat anti-mouse Siglec-F (E50-2440, 552126 BD Biosciences), Armenian hamster anti-mouse CD80 (16-10A1, 104729 Biolegend) and rabbit anti-mouse pNF-κB p65 (Ser536) (93H1,3033S Cell Signalling). After washing three times with PBST (0.1% Tween in PBS), the following secondary antibodies were added (1:400 in blocking solution) to the slides for 1 h at room temperature: AlexaFluor goat anti-rat 594 (A-11007), AlexaFluor goat anti-hamster 647 (A-21451) and AlexaFluor goat anti-rabbit 488 (A-11008), all from Thermo Fisher Scientific. Slides were washed four times for 5 min with PBST, and DAPI (D9542 Sigma, 1:1,000) was added to the third washing step. Slides were mounted in Prolog Gold (P36930 Invitrogen) and imaged on a Nikon Ti2-E inverted microscope, equipped with CrestOptics X-Light v3 confocal disk unit, Lumencor Celesta lasers and Photometrics Kinetix camera.

#### Human tissue microarrays

The microarrays CO245 and CO246 were obtained from TissueArray.Com. Deparaffinized sections were subjected to antigen retrieval in 2.4 mM sodium citrate and 1.6 mM citric acid, pH 6, for 25 min in a steamer. Sections were washed with PBST and blocked for 1 h at room temperature in blocking buffer (5% BSA, 5% heat-inactivated normal goat serum in PBST). Slides were incubated overnight at 4 °C with the following primary antibodies (1:100, in blocking buffer): mouse anti-human MBP (BMK-13, anti-human MBP (BMK-13, MCA5751 Bio-Rad) and rabbit anti-human PD-L1 (E1L3N, 13684S Cell Signalling). After washing three times with PBST (0.1% Tween in PBS), the following secondary antibodies were added (1:400 in blocking solution) to the slides for 1 h at room temperature: AlexaFluor goat anti-rabbit 594 and AlexaFluor goat anti-mouse 647 (Thermo Fisher Scientific). DAPI staining, mounting and imaging were performed as above.

#### Cytospins

A total of 10^5^ FACS-enriched spleen, blood and GI-tract-derived eosinophils (*Il5*-tg) from 7–8-week-old female and male mice were resuspended in 100 µl 5% FCS-supplemented RPMI medium and cytospun for 5 min at 50*g* into a funnel. Slides were air-dried for 30 min, fixed with ice-cold methanol for 5 min and then left to air dry overnight. Slides were washed, incubated for 1 h in blocking solution and stained overnight at 4 °C with mouse anti-EPX antibody (MM25-82.2.1 1:200, provided by E. A. Jacobsen), followed by 1-h incubation at room temperature with AlexaFluor goat anti-mouse 647. DAPI staining, mounting and imaging were performed as above. EPX staining intensity was quantified across the cell diameter in Fiji (MultiPlot) for 15 cells per condition.

### Image analysis for quantification of the active-to-basal ratio of eosinophils

The cores used for quantification as well as patient data are available in Supplementary Table 2. Cores were chosen on the basis of the presence of colonic epithelium. ND files were imported in Imaris 9.6.0 and spot objects were created in the green (MBP) and red (PD-L1) channels separately (estimated *XY* diameter = 7 µm, estimated *Z* diameter = 4 um, quality filter > 6). To quantify the co-expression of PD-L1 and MBP, the distance of each spot in the green channel to the nearest spot in the red channel was computed. Green spots (eosinophils) with distance to red spots < 4 µm were considered as active eosinophils (co-expressing PD-L1). Green spots with distance to red spots > 4 µm were considered basal eosinophils. The active-to-basal ratio was then computed by dividing the number of active by the number of basal eosinophils in each core. For localization analysis, the active-to-basal ratio in colon crypts of human and mouse tissue was calculated in manually drawn ROIs comprising the lower (basal) or upper (luminal) thirds.

### Histological assessment of colitis

Transversal mid-colon sections (0.5 cm) were fixed overnight in buffered 10% formalin solution, followed by paraffin embedding. Sections were stained with H&E. Histopathology of the colon was scored in a blinded manner considering four categories (each scored on a scale of 0–3): epithelial hyperplasia or damage and goblet cell depletion; leukocyte infiltration in the lamina propria; submucosal inflammation and oedema; area of tissue affected. The final score presented (0–12) represents the sums of all categories.

### In vitro genome-wide CRISPR inhibition screen

A total of 1.3 billion BM stem cells (BMSCs) from 10–16-week-old female and male mice (*n* = 27, dCas9-KRAB) were isolated as described above. BMSCs were then split in two replicates and each lentivirally transduced with an independently amplified genome-wide CRISPR inhibition library^57^ (Addgene 83987). Five days after transduction, BFP^+^ BMSCs were FACS-enriched and their culture medium was supplemented with recombinant IL-5 (10 ng ml^−1^, PeproTech). After six days of IL-5-mediated differentiation, BM-Eos were conditioned with colon CM overnight (1:10). PD-L1^+^CD80^+^ eosinophils were sorted, the genomic DNA was extracted and sgRNAs were target amplified. Library size distribution was measured with the Agilent high-sensitivity D5000 assay on a TapeStation 4200 system (5067–5592 Agilent Technologies). Sequencing was performed in single-end mode (75 cycles) on Illumina NextSeq. Reads were trimmed with cutadapt (ref. ^58^) and aligned to the sgRNA references with Bowtie2 (ref. ^59^). MAGeCK (ref. ^60^) was used for guide counting and paired testing.

### Western blotting

BM-Eos were isolated from 8–10-week-old female and male mice (B6J), differentiated and purified by flow cytometry. Cells were conditioned with colon CM (1:10) or rec-IL-33 (20 ng ml^−1^ PeproTech) for 45 min, then lysed in RIPA buffer (R0278 Sigma) supplemented with 2 mM sodium orthovanadate (J60191.AE Thermo Fisher Scientific), 15 mM sodium pyrophosphate (J62052.AK Thermo Fisher Scientific), 10 mM sodium fluoride (447351000 Thermo Fisher Scientific), and 1× complete protease inhibitor cocktail (11836153001 Roche). Protein concentrations were determined by BCA assay (23227 Pierce), and equal amounts were separated by SDS–PAGE using 10% acrylamide gels followed by transfer onto nitrocellulose membranes (88018 Thermo Fisher Scientific). Membranes were probed with antibodies against vinculin (42H89L44, 700062 Thermo Fisher Scientific), phospho-p38 MAPK (Thr180/Tyr182, MA5-15218 Thermo Fisher Scientific) and phospho-p65 (Ser536, 93H1, 3033 Cell Signalling Technology).

### Enzyme-linked immunosorbent assay (ELISA)

Proteins were extracted from colon samples homogenized in 450 μl RIPA lysis buffer (Thermo Fisher Scientific) supplemented with Na_3_Vo_4_ (100 mM), NaF (10 mM) and protease inhibitor cocktail (cOmplete, Mini Protease Inhibitor Tablets, 11836153001 Roche). The supernatant was collected and centrifuged at maximum speed for 10 min at 4 °C. Protein concentration was quantified with the Pierce BCA Protein Assay Kit (23225, Thermo Fisher Scientific). Plasma was isolated from blood in BD Microtainer tubes (365968, BD). Plates were coated overnight and the mouse IL-33 ELISA kit (88-7333-88 Thermo Fisher Scientific) was used to quantify the colon and plasma levels of IL-33 according to the manufacturer’s instructions.

### LEGENDplex bead-based immunoassay

Proteins were extracted as described above. Colon and plasma levels of IFNγ, IL-22 and TNF were quantified using LEGENDplex MU Th17 Panel (7-plex) according to the manufacturer’s instructions.

### Molecular cartography

#### Sample preparation

Fresh frozen colon samples from three patients with ulcerative colitis were sectioned onto coverslips and processed by Resolve Biosciences.

#### Segmentation

Cellpose (v. 2.0.4) (ref. ^61^) was used to segment nuclei in the DAPI images with the pretrained nuclei model and flow_threshold 0.5, cellprob_threshold −0.2. The nuclear segments were then expanded by 10 pixel (1.38 µm) using the ‘expand_labels’ function in scikit-image and transcripts were subsequently assigned to the expanded segments. Segments with fewer than three molecules or three genes detected were removed from the analysis.

#### Segmentation-free approach

To circumvent issues of segmentation, we used a transcript-focused approach in which we used spatial clusters of specific marker genes to represent cell types and investigate co-localization. For this, distances between individual transcripts of *CD4*, *SIGLEC8*, *CD8A*, *CD19*, *FOXP3* and *FCN1* were computed using Euclidean distances of the 2D coordinates. Hierarchical clustering was then applied to the distance matrix with average linkage to prevent chaining and a tree cut at height of 5 µm (hclust in the stats R package). We then used a k-d-tree based nearest neighbour search to identify the clusters in the surrounding area of each other cluster in a pre-defined radius of 10 µm as implemented in the R function ‘nn2’ (RANN v.2.6.1, searchtype=‘radius’) with a sufficiently large *k* (*k* = 41). This approach runs in O(M logM) time and avoids computation of a distance matrix for thousands of objects. Finally, a neighbourhood graph was constructed from the resulting adjacency matrix in which vertices (transcript clusters) are connected by edges if they are no further apart than 10 µm. From this graph the number of edges between different cell types was computed and compared to an empirical null distribution that was derived from randomly permuting the labels of the vertices (*m* = 1,000). This approach takes tissue composition and spatial structure into account and allows the computation of *P* values as *P* = (*b* + 1)/(*m* + 1), where *b* is the number of times the permutation produced a more extreme number of edges between two cell types than observed and *m* the total number of permutations^62^. This was done for each slide and possible cell–cell interaction to derive a score that represents the fraction of images in which a specific interaction was significant, with the sign representing interaction or avoidance; visualization was adopted from ref. ^63^.

### Graphical illustrations

Schematics of experimental workflows were created using a licensed version of Biorender.com.

### Reporting summary

Further information on research design is available in the Nature Portfolio Reporting Summary linked to this article.

## Online content

Any methods, additional references, Nature Portfolio reporting summaries, source data, extended data, supplementary information, acknowledgements, peer review information; details of author contributions and competing interests; and statements of data and code availability are available at 10.1038/s41586-022-05628-7.

## Supplementary information

Supplementary Information Supplementary Discussion and Supplementary References.

Reporting Summary

Supplementary Figure Uncropped western blot images.

Supplementary Figure Gating strategy used for eosinophil flow cytometric analysis and sorting.

Supplementary Table 1 List of subset markers. List of top 100 markers for each eosinophil subset generated with FindAllMarkers function in Seurat.

Supplementary Table 2 Patient information of TMA samples. Patient gender, age and pathology information.

Supplementary Table 3 Genes for signature. List of genes used to define scores and signatures, with respective references.

Supplementary Table 4 List of DEGs of bulk RNA-seq. Differentially expressed genes per condition.

Supplementary Table 5 Antibody list. List of antibodies (antigen, clone, fluorochrome, dilution and manufacturer) used for high-dimensional spectral flow cytometric analysis.

## Data Availability

Single-cell and bulk RNA-seq data generated during this study have been deposited at the Gene Expression Omnibus under the accession number GSE182001.

## Code availability

The code used in this study is available at https://github.com/Moors-Code/Eosinophils_scRNASeq.

## References

[1] Marichal T, Mesnil C, Bureau F (2017). Homeostatic eosinophils: characteristics and functions. Front. Med..

[2] Blanchard C, Wang N, Rothenberg ME (2006). Eosinophilic esophagitis: pathogenesis, genetics, and therapy. J. Allergy Clin. Immunol..

[3] Humbles AA (2004). A critical role for eosinophils in allergic airways remodeling. Science.

[4] Jenerowicz D, Czarnecka-Operacz M, Silny W (2007). Peripheral blood eosinophilia in atopic dermatitis. Acta Dermatovenerol. Alp Pannonica Adriat..

[5] Raab, Y., Fredens, K., Gerdin, B. & Hällgren, R. Eosinophil activation in ulcerative colitis: studies on mucosal release and localization of eosinophil granule constituents. *Dig. Dis. Sci*. **43**, 1061–1070 (1998). 10.1023/a:1018843104511 9590423

[6] Chu, V. T. et al. Eosinophils promote generation and maintenance of immunoglobulin-A-expressing plasma cells and contribute to gut immune homeostasis. *Immunity* **40**, 582–593 (2014). 10.1016/j.immuni.2014.02.014 24745334

[7] Jung Y (2015). IL-1β in eosinophil-mediated small intestinal homeostasis and IgA production. Mucosal Immunol..

[8] Ignacio, A. et al. Small intestinal resident eosinophils maintain gut homeostasis following microbial colonization. *Immunity* **55**, 1250–1267 (2022). 10.1016/j.immuni.2022.05.014 35709757

[9] Sugawara R (2016). Small intestinal eosinophils regulate Th17 cells by producing IL-1 receptor antagonist. J. Exp. Med..

[10] Alhmoud T (2020). Outcomes of inflammatory bowel disease in patients with eosinophil-predominant colonic inflammation. BMJ Open Gastroenterol..

[11] Smillie CS (2019). Intra- and inter-cellular rewiring of the human colon during ulcerative colitis. Cell.

[12] Sikkema, L. et al. An integrated cell atlas of the human lung in health and disease. Preprint at 10.1101/2022.03.10.483747 (2022).

[13] Lee NA (1997). Expression of IL-5 in thymocytes/T cells leads to the development of a massive eosinophilia, extramedullary eosinophilopoiesis, and unique histopathologies. J. Immunol..

[14] Mahmudi-Azer S, Downey GP, Moqbel R (2002). Translocation of the tetraspanin CD63 in association with human eosinophil mediator release. Blood.

[15] Khushman M (2019). Exosomal markers (CD63 and CD9) expression and their prognostic significance using immunohistochemistry in patients with pancreatic ductal adenocarcinoma. J. Gastrointest. Oncol..

[16] Cohnen A (2013). Surface CD107a/LAMP-1 protects natural killer cells from degranulation-associated damage. Blood.

[17] Mesnil C (2016). Lung-resident eosinophils represent a distinct regulatory eosinophil subset. J. Clin. Invest..

[18] Schwarzfischer M (2022). TiO_2_ nanoparticles abrogate the protective effect of the Crohn’s disease-associated variation within the PTPN22 gene locus. Gut.

[19] Qiu X (2017). Reversed graph embedding resolves complex single-cell trajectories. Nat. Methods.

[20] Bergen V, Lange M, Peidli S, Wolf FA, Theis FJ (2020). Generalizing RNA velocity to transient cell states through dynamical modeling. Nat. Biotechnol..

[21] Lange M (2022). CellRank for directed single-cell fate mapping. Nat. Methods.

[22] McFaline-Figueroa JL (2019). A pooled single-cell genetic screen identifies regulatory checkpoints in the continuum of the epithelial-to-mesenchymal transition. Nat. Genet..

[23] Arnold IC (2018). Eosinophils suppress Th1 responses and restrict bacterially induced gastrointestinal inflammation. J. Exp. Med..

[24] Efremova M, Vento-Tormo M, Teichmann SA, Vento-Tormo R (2020). CellPhoneDB: inferring cell–cell communication from combined expression of multi-subunit ligand–receptor complexes. Nat. Protoc..

[25] Masterson JC (2015). Eosinophil-mediated signalling attenuates inflammatory responses in experimental colitis. Gut.

[26] Arnold, I. C. et al. The GM–CSF–IRF5 signaling axis in eosinophils promotes antitumor immunity through activation of type 1 T cell responses. *J. Exp. Med.* **217**, e20190706 (2020). 10.1084/jem.20190706 PMC7953737 32970801

[27] Griseri T (2015). Granulocyte macrophage colony-stimulating factor-activated eosinophils promote interleukin-23 driven chronic colitis. Immunity.

[28] Aibar S (2017). SCENIC: single-cell regulatory network inference and clustering. Nat. Methods.

[29] Griesenauer B, Paczesny S (2017). The ST2/IL-33 axis in immune cells during inflammatory diseases. Front. Immunol..

[30] Kang K (2017). Interferon-γ represses M2 gene expression in human macrophages by disassembling enhancers bound by the transcription factor MAF. Immunity.

[31] Menzella, F. et al. Anti-IL5 therapies for severe eosinophilic asthma: literature review and practical insights. *J. Asthma Allergy* **13**, 301–313 (2020). 10.2147/JAA.S258594 PMC7490042 32982318

[32] Adachi O (1998). Targeted disruption of the *MyD88* gene results in loss of IL-1-and IL-18-mediated function. Immunity.

[33] Hoshino K (1999). Cutting edge: Toll-like receptor 4 (TLR4)-deficient mice are hyporesponsive to lipopolysaccharide: evidence for TLR4 as the *Lps* gene product. J. Immunol..

[34] Rawlins EL, Clark CP, Xue Y, Hogan BLM (2009). The Id2^+^ distal tip lung epithelium contains individual multipotent embryonic progenitor cells. Development.

[35] Dent LA, Strath M, Mellor AL, Sanderson CJ (1990). Eosinophilia in transgenic mice expressing interleukin 5. J. Exp. Med..

[36] Lee H-M (2015). IFNγ signaling endows DCs with the capacity to control type I inflammation during parasitic infection through promoting T-bet^+^ regulatory T cells. PLoS Pathog..

[37] Oboki K (2010). IL-33 is a crucial amplifier of innate rather than acquired immunity. Proc. Natl Acad. Sci. USA..

[38] Townsend MJ, Fallon PG, Matthews DJ, Jolin HE, McKenzie AN (2000). T1/St2-deficient mice demonstrate the importance of T1/St2 in developing primary T helper cell type 2 responses. J. Exp. Med..

[39] Lee JJ (2004). Defining a link with asthma in mice congenitally deficient in eosinophils. Science.

[40] Doyle AD (2013). Homologous recombination into the eosinophil peroxidase locus generates a strain of mice expressing Cre recombinase exclusively in eosinophils. J. Leukoc. Biol..

[41] Diehl GE (2013). Microbiota restricts trafficking of bacteria to mesenteric lymph nodes by CX_3_CR1^hi^ cells. Nature.

[42] Wiles S, Pickard KM, Peng K, MacDonald TT, Frankel G (2006). In vivo bioluminescence imaging of the murine pathogen *Citrobacter rodentium*. Infect. Immun..

[43] Hao Y (2021). Integrated analysis of multimodal single-cell data. Cell.

[44] Korsunsky I (2019). Fast, sensitive and accurate integration of single-cell data with Harmony. Nat. Methods.

[45] Kowalczyk MS (2015). Single-cell RNA-seq reveals changes in cell cycle and differentiation programs upon aging of hematopoietic stem cells. Genome Res..

[46] Trapnell C (2014). The dynamics and regulators of cell fate decisions are revealed by pseudotemporal ordering of single cells. Nat. Biotechnol..

[47] Cacchiarelli D (2018). Aligning single-cell developmental and reprogramming trajectories identifies molecular determinants of myogenic reprogramming outcome. Cell Syst.

[48] La Manno G (2018). RNA velocity of single cells. Nature.

[49] Holland CH (2020). Robustness and applicability of transcription factor and pathway analysis tools on single-cell RNA-seq data. Genome Biol..

[50] Gu Z, Eils R, Schlesner M (2016). Complex heatmaps reveal patterns and correlations in multidimensional genomic data. Bioinformatics.

[51] Wickham, H. *ggplot2: Elegant Graphics for Data Analysis* (Springer, 2016).

[52] Brummelman J (2019). Development, application and computational analysis of high-dimensional fluorescent antibody panels for single-cell flow cytometry. Nat. Protoc..

[53] Dyer, K. D. et al. Functionally competent eosinophils differentiated ex vivo in high purity from normal mouse bone marrow. *J. Immunol.* **181**, 4004–4009 (2008). 10.4049/jimmunol.181.6.4004 PMC2680436 18768855

[54] Hatakeyama M (2016). SUSHI: an exquisite recipe for fully documented, reproducible and reusable NGS data analysis. BMC Bioinformatics.

[55] Robinson MD, McCarthy DJ, Smyth G (2010). edgeR: a Bioconductor package for differential expression analysis of digital gene expression data. Bioinformatics.

[56] Kolde, R. pheatmap: Pretty Heatmaps. R version 1.0.12, https://cran.r-project.org/web/packages/pheatmap/index.html (2019).

[57] Horlbeck MA (2016). Compact and highly active next-generation libraries for CRISPR-mediated gene repression and activation. eLife.

[58] Martin M (2011). Cutadapt removes adapter sequences from high-throughput sequencing reads. EMBnet.J.

[59] Langmead B, Salzberg SL (2012). Fast gapped-read alignment with Bowtie 2. Nat. Methods.

[60] Li W (2014). MAGeCK enables robust identification of essential genes from genome-scale CRISPR/Cas9 knockout screens. Genome Biol..

[61] Stringer C, Wang T, Michaelos M, Pachitariu M (2021). Cellpose: a generalist algorithm for cellular segmentation. Nat. Methods.

[62] Phipson B, Smyth GK (2010). Permutation P-values should never be zero: calculating exact P-values when permutations are randomly drawn. Stat. Appl. Genet. Mol. Biol..

[63] Lohoff T (2022). Integration of spatial and single-cell transcriptomic data elucidates mouse organogenesis. Nat. Biotechnol..

