# Supplementary material for: Active eosinophils regulate host defence and immune responses in colitis
Source: Nature. 2022 Dec 12;615(7950):151–7. doi: 10.1038/s41586-022-05628-7 (PMC9977678; doi:10.1038/s41586-022-05628-7)
Supplement: Supplementary file 1 — Supplementary Discussion and Supplementary References. [file 41586_2022_5628_MOESM1_ESM.pdf]

nature portfolio

https://doi.org/10.1038/s41586-022-05628-7

Supplementary information

Active eosinophils regulate host defence and immune responses in colitis

In the format provided by the authors and unedited

Supplementary Discussion

The extended survival and high numbers of eosinophils present in the GI tract at steady state, and their accumulation during infection and inflammation, suggest eosinophils play cardinal roles in intestinal homeostatic and inflammatory processes. Here, we investigated the existence and ontogenetic relationship of eosinophil subsets to define their functional contribution to intestinal health and disease. We identified five consecutive developmental and maturation stages of the eosinophil lineage: (i) precursors in the BM, the only proliferative stage, (ii) immature eosinophils, characterized by granule protein production and present in the BM and blood, (iii) circulating eosinophils that migrate to target tissues, B-Eos, a tissue-resident population shared across peripheral tissues and (v) A-Eos, a subset mostly specific to the GI tract, characterized by PD-L1 and CD80 expression, bactericidal activity, T-cell regulatory and tissue-protective function during acute inflammation.

Our findings suggest that eosinophil subpopulations emerge from consecutive maturation steps and adaptation to distinct niches. This observation corroborates proposals that the considerable morphological, phenotypic and transcriptional variability of tissue eosinophils reflects their adaptation to different microenvironments1-3. While GI A-Eos shared a common transcriptional signature with other tissue resident eosinophils such as those from the uterus, lung or adipose, this subset also exhibited a clearly distinct gene expression profile as a result of their GI tissue maturation. Indeed, A-Eos upregulated a multitude of surface receptors, suggesting this subset responds and readily adapts to local microbial, tissue and cytokine cues. The residence of A-Eos in the luminal third of the intestinal mucosa coincides with the activation of HiF1-a, which may allow their adaptation to the oxygen gradient that decreases steeply from the intestinal submucosa to the anaerobic lumen[4]. The presence of this subset close to the intestinal epithelium is consistent with a $CD11c^{hi}$ eosinophil population reported to localize to the intraepithelial fraction of the SI[5]. Thus, our study expands the concept of myeloid cell plasticity to the eosinophil lineage, a notion so far only explored thoroughly for macrophages[6] and neutrophils[7].

The upregulation of multiple enzyme, cytokine and chemokine transcripts in A-Eos further indicates a marked increase in biosynthetic activity upon differentiation of this subset. Although eosinophil cytokines are predominantly found preformed and stored within their granules and secretory vesicles[8], *de novo* production of these factors in A-Eos suggests they are strictly required to fulfill tissue-specific activities. Among the signature genes of this

cluster, *Vegfa, Il1b* and *Il1rn* have been linked to eosinophil-mediated angiogenesis[9], support of intestinal homeostasis and IgA production[10,11] as well as downmodulation of local Th17 responses[12], respectively.

Our study further highlights the prevalent expression of T cell co-stimulatory molecules by B-Eos (*Icosl*) and, more predominantly, by A-Eos (*Cd9*, *Cd80*, *Cd274*) and identifies CD80 and PD-L1 as *bona fide* markers of both the human and mouse A-Eos subset. We indeed show that eosinophils enhance T cell proliferation in their basal state, but downmodulate this activity following intestinal conditioning or IL-33 exposure, particularly in conjunction with IFN-γ. A-Eos might thus control the extent of $CD4^+$ T cell responses, potentially via co-stimulatory molecule expression, to limit tissue damage during Th1 inflammation. These observations are consistent with our previous report indicating a functional role for PD-L1 and IFN-γ signaling in eosinophil-mediated T cell suppression[13] and corroborate studies supporting the regulation of T cell activities by GI eosinophils[12,14,15]. Further functional characterization of the eosinophil co-stimulatory markers identified in this study is warranted.

Among the signals driving A-Eos differentiation, we identified microbially-derived cues, IL-33 and IFN-γ as critical drivers of their adaptation *in situ*. A-Eos maturation required microbial presence but was independent of major bacterial recognition pathways (TLR2/4), suggesting that microbiota-derived signals may act on eosinophils via alternative bacterial sensors or indirectly via the epithelium or immune cells. Furthermore, the alarmin IL-33, but not other cytokines such as IL-22 and IL-25, was sufficient to induce A-Eos maturation. IL-33 is known to be involved in eosinophilic disorders[16] and signalling via ST2 receptor was recently reported to promote eosinophil homeostatic functions in the small intestine in a microbiota-dependent manner[17]. Our data show that IL-33 signalling via the ST2/MyD88 axis is required for A-Eos accumulation during colitis and that it promotes their ability to downregulate T cell responses. This effect was further reinforced by IFN-γ signaling, which promoted PD-L1 upregulation and the induction of genes involved in antigen presentation. In addition, IFN-γ stimulation induced a shift in the transcriptome of BM-Eos to a more mature state and promoted the mobilisation and focal aggregation of EPX granular proteins at the cellular periphery. These observations are in line with previous reports indicating a role for IFN-γ in inducing a transcriptional signature resembling that of eosinophils during DSS colitis[18], in promoting eosinophil primary granule mobilisation and piecemeal degranulation *in vitro*[19], as well as in enhancing EPX degranulation and eosinophil bactericidal activity *in vivo*[13].

An intriguing new finding of our study is the striking compositional shift toward the active cluster at sites of infection and inflammation. The absence of new eosinophil cluster(s) arising during bacterial infection and colitis, as observed in three independent models, suggests that eosinophils recruited in this context do not differ substantially from their steady state counterparts, but rather acquire new functional properties, largely driven by local interferon and cytokine signaling networks (Irf1/2/7/9; Stat1/3-6; NFkb1/2, Rela/b). This finding contrast with a previous report indicating that inflammatory eosinophils accumulating during allergen challenge in the lung differ morphologically, phenotypically and functionally from lung-resident eosinophils[20]. Interestingly, the sustained expression of antimicrobial peptides and cytotoxic granule protein genes was already detectable during eosinophil maturation in the BM and blood. Together with the bypassing of certain maturity stages such as the basal state, this may ensure the rapid deployment of "primed" eosinophils at sites of bacterial invasion. The appearance of EPX-rich cellular protrusions in A-Eos following bacterial challenge is consistent with our previous report showing that eosinophils undergo degranulation and EETs release in response to *C. rod*, thus promoting bacterial clearance and host protection.[1312] This "non-lethal" mechanism of defense[21] may thus result in the formation of a tissue-protective scaffold preventing luminal bacterial translocation upon acute epithelial injury[22]. The presence of a PD-L1^{+} eosinophil subset in the normal human mucosa, and its accumulation during IBD, suggests the existence of a human counterpart of the A-Eos subset that may share its functional properties. Our data indicate that A-Eos are strongly enriched in CD, but less so in UC patients, and predominantly localize in proximity to CD4^{+} T cells, where they show evidence of IFN-γ signaling activity. While this observation raises the possibility that A-Eos attenuate T cell responses during intestinal inflammation in human, the superior secretory activity and cytotoxic potential associated with this subset may at the same time promote the pathological features of IBD over time through chronic tissue degradation. The effect of eosinophils on IBD outcomes is indeed unclear and typically associated with both, tissue protective and damaging activities[23-27]. As the severe mucosal damage characteristic of IBD also requires efficient wound healing activity, it remains to be determined how long-term imbalance in the A-to-B-Eos ratio affects these distinct processes during the active and remission phases of disease.

Overall, our study further elucidates the ontogeny of intestinal eosinophils and reveals the marked transcriptomic changes that occur during eosinophil maturation across anatomical compartments. The gene programs upregulated by eosinophils in the healthy and inflamed GI tract further highlight the major contribution of these cells to intestinal homeostasis, immune

regulation and host defense. Our findings provide novel insights into the biology of this elusive cell type and lay a framework for the functional characterization of eosinophil subsets in GI diseases.

References

1 Abdala-Valencia, H. *et al.* Shaping eosinophil identity in the tissue contexts of development, homeostasis, and disease. *J Leukoc Biol* **104**, 95-108, doi:10.1002/JLB.1MR1117-442RR (2018).

2 Masterson, J. C., Menard-Katcher, C., Larsen, L. D., Furuta, G. T. & Spencer, L. A. Heterogeneity of Intestinal Tissue Eosinophils: Potential Considerations for Next-Generation Eosinophil-Targeting Strategies. *Cells* **10**, doi:10.3390/cells10020426 (2021).

3 Diny, N. L. *et al.* The aryl hydrocarbon receptor contributes to tissue adaptation of intestinal eosinophils in mice. *J Exp Med* **219**, doi:10.1084/jem.20210970 (2022).

4 Zheng, L., Kelly, C. J. & Colgan, S. P. Physiologic hypoxia and oxygen homeostasis in the healthy intestine. A Review in the Theme: Cellular Responses to Hypoxia. *Am J Physiol Cell Physiol* **309**, C350-360, doi:10.1152/ajpcell.00191.2015 (2015).

5 Xenakis, J. J. *et al.* Resident intestinal eosinophils constitutively express antigen presentation markers and include two phenotypically distinct subsets of eosinophils. *Immunology* **154**, 298-308, doi:10.1111/imm.12885 (2018).

6 Lavin, Y. *et al.* Tissue-resident macrophage enhancer landscapes are shaped by the local microenvironment. *Cell* **159**, 1312-1326, doi:10.1016/j.cell.2014.11.018 (2014).

7 Ballesteros, I. *et al.* Co-option of Neutrophil Fates by Tissue Environments. *Cell* **183**, 1282-1297 e1218, doi:10.1016/j.cell.2020.10.003 (2020).

8 Melo, R. C., Liu, L., Xenakis, J. J. & Spencer, L. A. Eosinophil-derived cytokines in health and disease: unraveling novel mechanisms of selective secretion. *Allergy* **68**, 274-284, doi:10.1111/all.12103 (2013).

9 Hoshino, M., Nakamura, Y. & Hamid, Q. A. Gene expression of vascular endothelial growth factor and its receptors and angiogenesis in bronchial asthma. *J Allergy Clin Immunol* **107**, 1034-1038, doi:10.1067/mai.2001.115626 (2001).

10 Jung, Y. *et al.* IL-1beta in eosinophil-mediated small intestinal homeostasis and IgA production. *Mucosal Immunol* **8**, 930-942, doi:10.1038/mi.2014.123 (2015).

11 Chu, V. T. *et al.* Eosinophils promote generation and maintenance of immunoglobulin-A-expressing plasma cells and contribute to gut immune homeostasis. *Immunity* **40**, 582-593, doi:10.1016/j.immuni.2014.02.014 (2014).

12 Sugawara, R. *et al.* Small intestinal eosinophils regulate Th17 cells by producing IL-1 receptor antagonist. *J Exp Med* **213**, 555-567, doi:10.1084/jem.20141388 (2016).

13 Arnold, I. C. *et al.* Eosinophils suppress Th1 responses and restrict bacterially induced gastrointestinal inflammation. *J Exp Med* **215**, 2055-2072, doi:10.1084/jem.20172049 (2018).

14 Arnold, I. C. *et al.* The GM-CSF-IRF5 signaling axis in eosinophils promotes antitumor immunity through activation of type 1 T cell responses. *J Exp Med* **217**, doi:10.1084/jem.20190706 (2020).

15 Fallegger, A. *et al.* TGF-beta production by eosinophils drives the expansion of peripherally induced neuropilin(-) RORgammat(+) regulatory T-cells during bacterial and allergen challenge. *Mucosal Immunol*, doi:10.1038/s41385-022-00484-0 (2022).

16 K. Shah, A. I., J. Bernier-Latmani, Y. Köller, G. Coakley, M. Moyat, R. Hamelin, F. Armand, N. C. Wong, H. Ramay, C.A. Thomson, R. Burkhard, A. Dufour, T.V. Petrova, N.L. Harris, K. D. McCoy. Small Intestinal Resident Eosinophils Maintain Gut Homeostasis Following Microbial Colonisation. *bioRxiv Preprint*, doi:10.1101/2021.01.30.428930 (2021).

17 Ignacio, A. *et al.* Small intestinal resident eosinophils maintain gut homeostasis following microbial colonization. *Immunity* **55**, 1250-1267 e1212, doi:10.1016/j.immuni.2022.05.014 (2022).

18 Dolitzky, A. *et al.* Transcriptional Profiling of Mouse Eosinophils Identifies Distinct Gene Signatures Following Cellular Activation. *Front Immunol* **12**, 802839, doi:10.3389/fimmu.2021.802839 (2021).

19 Lacy, P. *et al.* Rapid mobilization of intracellularly stored RANTES in response to interferon-gamma in human eosinophils. *Blood* **94**, 23-32 (1999).

20 Mesnil, C. *et al.* Lung-resident eosinophils represent a distinct regulatory eosinophil subset. *J Clin Invest* **126**, 3279-3295, doi:10.1172/JCI85664 (2016).

21 Yousefi, S. *et al.* Catapult-like release of mitochondrial DNA by eosinophils contributes to antibacterial defense. *Nat Med* **14**, 949-953, doi:10.1038/nm.1855 (2008).

22 Gigon, L., Yousefi, S., Karaulov, A. & Simon, H. U. Mechanisms of toxicity mediated by neutrophil and eosinophil granule proteins. *Allergol Int* **70**, 30-38, doi:10.1016/j.alit.2020.11.003 (2021).

23 Raab, Y., Fredens, K., Gerdin, B. & Hallgren, R. Eosinophil activation in ulcerative colitis: studies on mucosal release and localization of eosinophil granule constituents. *Dig Dis Sci* **43**, 1061-1070, doi:10.1023/a:1018843104511 (1998).

24 Saitoh, O. *et al.* Fecal eosinophil granule-derived proteins reflect disease activity in inflammatory bowel disease. *Am J Gastroenterol* **94**, 3513-3520, doi:10.1111/j.1572-0241.1999.01640.x (1999).

25 Lampinen, M. *et al.* Eosinophil granulocytes are activated during the remission phase of ulcerative colitis. *Gut* **54**, 1714-1720, doi:10.1136/gut.2005.066423 (2005).

26 Gabriels, R. Y. *et al.* Mucosal Eosinophil Abundance in Non-Inflamed Colonic Tissue Is Associated with Response to Vedolizumab Induction Therapy in Inflammatory Bowel Disease. *J Clin Med* **11**, doi:10.3390/jcm11144141 (2022).

27 Alhmoud, T. *et al.* Outcomes of inflammatory bowel disease in patients with eosinophil-predominant colonic inflammation. *BMJ Open Gastroenterol* **7**, e000373, doi:10.1136/bmjgast-2020-000373 (2020).
