# Supplementary material for: Active eosinophils regulate host defence and immune responses in colitis
Source: Nature. 2022 Dec 12;615(7950):151–7. doi: 10.1038/s41586-022-05628-7 (PMC9977678; doi:10.1038/s41586-022-05628-7)
Supplement: Supplementary file 2 — Reporting Summary [file 41586_2022_5628_MOESM2_ESM.pdf]

nature portfolio

Corresponding author(s): Isabelle C. Arnold, Andreas E. Moor

Last updated by author(s): Nov 14, 2022

Reporting Summary

Nature Portfolio wishes to improve the reproducibility of the work that we publish. This form provides structure for consistency and transparency in reporting. For further information on Nature Portfolio policies, see our Editorial Policies and the Editorial Policy Checklist.

Statistics

For all statistical analyses, confirm that the following items are present in the figure legend, table legend, main text, or Methods section.

n/a	Confirmed	
☐	☒	The exact sample size (*n*) for each experimental group/condition, given as a discrete number and unit of measurement
☐	☒	A statement on whether measurements were taken from distinct samples or whether the same sample was measured repeatedly
☐	☒	The statistical test(s) used AND whether they are one- or two-sided<br>*Only common tests should be described solely by name; describe more complex techniques in the Methods section.*
☒	☐	A description of all covariates tested
☐	☒	A description of any assumptions or corrections, such as tests of normality and adjustment for multiple comparisons
☐	☒	A full description of the statistical parameters including central tendency (e.g. means) or other basic estimates (e.g. regression coefficient) AND variation (e.g. standard deviation) or associated estimates of uncertainty (e.g. confidence intervals)
☐	☒	For null hypothesis testing, the test statistic (e.g. *F*, *t*, *r*) with confidence intervals, effect sizes, degrees of freedom and *P* value noted<br>*Give P values as exact values whenever suitable.*
☒	☐	For Bayesian analysis, information on the choice of priors and Markov chain Monte Carlo settings
☒	☐	For hierarchical and complex designs, identification of the appropriate level for tests and full reporting of outcomes
☒	☐	Estimates of effect sizes (e.g. Cohen's *d*, Pearson's *r*), indicating how they were calculated

Our web collection on statistics for biologists contains articles on many of the points above.

Software and code

Policy information about availability of computer code

Data collection	No software was used.
Data analysis	GraphPad Prism v9.1.1 GraphPad Software Schneider https://www.graphpad.com/scientific- software/prism/<br>R software v3.6.3 or v4.1.0 GNU project https://www.r-project.org<br>R Studio Server v1.4.1717 https://www.rstudio.com<br>Bcl2fastq v2.20.0.422 (Illumina https://support.illumina.com/sequencing/sequencing_software/bcl2fastq-conversion-software.html)<br>Seven Bridges Genomics (BD Biosciences, https://www.sevenbridges.com)<br>edgeR v3.36.0 R package Robinson et al, 2010 https://bioconductor.org/packages/release/bioc/html/edgeR.html<br>pheatmap v1.0.12 R package Kolde, 2012 https://cran.r-project.org/web/packages/pheatmap/pheatmap.pdf<br>Seurat v4.03 Hao et al, 2021 https://satijalab.org/seurat/get_ started.html RRID:SCR_016341<br>msigdbr v7.5.1 R package R Bioconductor https://cran.r-project.org/web/packages/msigdbr/vignettes/msigdbr-intro.html<br>fgsea v1.20.0 R package Sergushichev et al, 2016 https://bioconductor.org/packages/release/bioc/html/fgsea.html<br>ggplot2 v3.3.6 R package Wickham, 2016 https://cloud.r-project.org/web/packages/ggplot2/index.html<br>monocle v2.3.6 R package, Trapnell et al, 2014<br>CellPhoneDB v2.0.0 R package, Efremova et al, 2020<br>ComplexHeatmap v2.10.0 R package Gu et al, 2016<br>SCENIC v1.2.4 R package, Aibar et al, 2017<br>PROGENy v1.13.2 R package, Holland et al, 2020<br>Biomart v2.50.3 R package v2.46.3<br>kplots v1.1.14 R package<br>FlowSOM v3.16 R package, Brummelman et al, 2019<br>Image J v2.0.0 Fiji Schindelin et al, 2012 https://imagej.net/Fiji/<br>FlowJo v10.7.1 (Becton Dickinson & Company)

BD FACSDiva Software v8.0.2 (BD Biosciences)
cutadapt v4.1, Martin et al 2011
Bowtie2 v2.5.0, Langmead et al, 2012
MAGeCK v0.5.9, Li et al, 2014
velocyto v0.17.16, La Manno et al, 2018
scvelo v0.2.0, Bergen et a, 2020
Cellrank v1.5.1, Lange et al, 2022
SUSHI framework, FGCZ, Hatekeyama et al, 2016
Cellpose, v2.0.4, Stringer et al, 2021
stats, v4.3.0, R package R Core Team, 2013
Code is available at https://github.com/Moors-Code/Eosinophils_scRNASeq

For manuscripts utilizing custom algorithms or software that are central to the research but not yet described in published literature, software must be made available to editors and reviewers. We strongly encourage code deposition in a community repository (e.g. GitHub). See the Nature Portfolio guidelines for submitting code & software for further information.

Data

Policy information about availability of data

All manuscripts must include a data availability statement. This statement should provide the following information, where applicable:

- Accession codes, unique identifiers, or web links for publicly available datasets
- A description of any restrictions on data availability
- For clinical datasets or third party data, please ensure that the statement adheres to our policy

ScRNA-seq data generated during this study are deposited at the Gene Expression Omnibus under access number GSE182001. Gene Ontology databases were downloaded through the R package msigdbr.

Field-specific reporting

Please select the one below that is the best fit for your research. If you are not sure, read the appropriate sections before making your selection.

☒ Life sciences ☐ Behavioural & social sciences ☐ Ecological, evolutionary & environmental sciences

For a reference copy of the document with all sections, see nature.com/documents/nr-reporting-summary-flat.pdf

Life sciences study design

All studies must disclose on these points even when the disclosure is negative.

Sample size	In accordance with the 3Rs, the smallest sample size was chosen that could give a significant difference. Given the robustness of the phenotypes across all methods used (transcriptome and protein level), the minimum sample size assuming no overlap in control versus experimental is three animals per experiment.
Data exclusions	No animals were excluded, unless data acquisition quality was insufficient.
Replication	Mouse experimental data was combined from independent experiments (at least 3 mice) with treated on different days and analyzed together. Micrographs of murine and human colon as well as cytospins are representative of at least two independent experiments. The results were consistent in all independent experiments.
Randomization	All experiments were performed on 6-16 week-old male and female mice, without separation between experimental groups and littermate controls. All samples were analyzed equally without sub-sampling, hence no randomization was required.
Blinding	The researcher was blinded to the genotype during the processing and analysis.

Reporting for specific materials, systems and methods

We require information from authors about some types of materials, experimental systems and methods used in many studies. Here, indicate whether each material, system or method listed is relevant to your study. If you are not sure if a list item applies to your research, read the appropriate section before selecting a response.

Materials & experimental systems

n/a	Involved in the study
☐	☒ Antibodies
☒	☐ Eukaryotic cell lines
☒	☐ Palaeontology and archaeology
☐	☒ Animals and other organisms
☒	☐ Human research participants
☒	☐ Clinical data
☒	☐ Dual use research of concern

Methods

n/a	Involved in the study
☒	☐ ChIP-seq
☐	☒ Flow cytometry
☒	☐ MRI-based neuroimaging

Antibodies

Antibodies used

For surface staining, cell were stained with fixable viability dye eFluor 780 (1:1000, 65-0865-14 eBioscience) and a combination of the following antibodies (1:200, all from BioLegend unless stated otherwise): anti-mouse CD45 BV650 (30-F11, 103151), CD11b BV510 (M1/70, 101263), MHC-II AF700 (M5/114.15.2, 107622), Ly6G Percp-Cy5.5 (1A8, 127616), CD4 PerCP (RM4-5, 100538), TCRβ PE-Cy7 (H57-597, 109222), TCRβ PE-Cy7 (H57-597, 109228), CD80 BV605 (1:100, 16-10A1, 104729), PD-L1 PE-Cy7 (1:100, 10F.9G2, 124314), CD31 PE (390, 102408), CD45.2 BV785 (1:50, 104, 109839), CD9 PE (MZ3, 124805), CD54 BV711 (YN1/1.7.4, 116143), CD63 PE (1:100, NVG-2, 143904), CD95 PE-Cy7(SA367H8, 152607), SiglecE PE (M1304A01, 677104), Sca-1 AF488 (D7, 108116), Sca-1 AF700 (D7, 108142), C-kit BV605 (ACK2, 135121), CD11c APC-Cy7 (N418, 117323), Clec12a PE (5D3, 143404), CD49d FITC (R1-2, 103605), CD16/32 FITC (S17012B, 101305), CD3e Percp-Cy5.5 (145-2C11, 100328), CD8a APC (53-6.7, 100712), NK1.1 Percp-Cy5.5 (PK136, 108727), B220 Percp-Cy5.5 (RA3-6B2, 103236), Ter119 Percp (TER-119, 116227), Gr1 Percp (RB6-8C5, 108427), CD34 AF647 (RAM34, 560230), Siglec F BV421 (E50-2440, 552681 BD Biosciences), Siglec F PE (E50-2440, 552126 BD Biosciences), CD125 PE (T21, 558488 BD Biosciences), CD275 (HK5.3, 50598582 eBioscience), T1/ST2 FITC (1:100, DJ8, 101001F MD Bioproductos GmbH). Fc block (anti-CD16/CD32, 101302 Affymetrix) was included to minimize nonspecific antibody binding.

For T cell intracellular cytokine staining: anti-mouse IL-17A APC (TC11-18H10.1, 506916), IFN-ɣ BV421 (XMG1.2, 505830) and TNF-α FITC (MP6-XT22, 506 304) all from Biolegend.

Neutralizing antibodies: anti-IL-33 neutralising antibody (AF3626, Biotechne), anti-IL-5 (BE0198 BioXCell, TREK5), anti keyhole limpet hemocyanin isotype control (BE0090, BioXCell, LTF-2), anti-IFN-ɣR (BE0029, BioXCell, GR-20), anti-CCR3 (BE0316 clone 6S2-19-49), anti-horseradish peroxidase isotype control (BE0088, BioXCell, HRPN).

For high-dimensional spectral flow-cytometry analysis see Table S4.

For Western blotting membranes were probed with antibodies (1:1000) against vinculin (42H89L44, 700062 Thermo Fisher Scientific), phospho-p38 MAPK (Thr180/Tyr182, MA5-15218 Thermo Fisher Scientific) and phospho-p65 (Ser536, 93H1, 3033 Cell Signalling Technology).

For immunohistochemistry: rat anti-mouse SiglecF (E50-2440, 552126 BD Biosciences), Armenian hamster anti-mouse CD80 (16-10A1, 104729 Biolegend), rabbit anti-mouse p-NF-KB p65 (Ser536) (93H1,3033S Cell Signalling), mouse anti-human MBP (BMK-13, anti-human MBP (BMK-13, MCA5751 Bio-RAD), rabbit anti-human PD-L1 (E1L3N, 13684S Cell Signalling), mouse anti-EPX antibody (MM25-82.2.1, kindly provided by Dr. E.A. Jacobsen from Mayo Clinic, Scottsdale, AZ). Secondary antibodies (ThermoFisher): AlexaFluor goat-anti hamster 647, AlexaFluor goat anti-rat 594 (A-11007), AlexaFluor goat-anti hamster 647 (A-21451), AlexaFluor goat anti-rabbit 488 (A-11008), AlexaFluor goat anti-mouse 647 (A-21235).

Validation

All antibodies have been previously validated extensively by the manufacturer including by Western blot and confirmed by the authors for specificity and localization (no primary antibody control for IF and FMO for flow cytometry).

Animals and other organisms

Policy information about studies involving animals; ARRIVE guidelines recommended for reporting animal research

Laboratory animals

All experiments were performed on 6-16 week-old male and female mice. C57BL/6J (B6J, stock no. 000664), and dCas9-KRAB (stock no.030000) mice were obtained from The Jackson Laboratory; OT-1 (stock no. 003831), OT-II (stock no. 004194), MyD88-/- (Adachi et al, 1998), Tlr2-/- (stock no. 004650), CD45.1 (stock no.002014), Tlr4-/- mice (Hoshino et al, 1999) were obtained from a local live mouse repository. Id2CreERT2;Rosa26EYFP mice (Rawlins et al, 2019), Il5–transgenic mice (Dent et al, 1990) and Ifngr2fl/fl mice Lee et al, 2015) have been previously described. Il33−/− mice (Oboki et al, 2010) were obtained through the RIKEN Center for Developmental Biology (Acc.No.CDB0631K) and St2−/− mice have been described (Townsend et al, 2000) and backcrossed onto a C57BL/6J background. Eosinophil-deficient mice (PHIL, Lee et al, 2004) and mice expressing Cre under the EPX promoter (Eo-Cre, Doyle et al, 2013) were obtained from J.J. Lee (Mayo Clinic, Phoenix, AZ). Mice were maintained in a specific-pathogen-free (SPF) facility with a 12-h light-dark cycle, under controlled temperature (18-23°C) and humidity (40-60%), with ad libitum standard diet and water.

Wild animals

No wild animals were used in this study.

Field-collected samples

No field collected samples were used in this study.

Ethics oversight

All experimental procedures at the University of Zurich and Bern were performed in accordance with Swiss Federal regulations and approved by the Cantonal Veterinary Office and/or in accordance with the European Communities Council Directive (86/609/EEC),

Czech national guidelines, institutional guidelines of the Institute of Molecular Genetics and approved by the Animal Care Committee.

Note that full information on the approval of the study protocol must also be provided in the manuscript.

Flow Cytometry

Plots

Confirm that:

☒ The axis labels state the marker and fluorochrome used (e.g. CD4-FITC).

☒ The axis scales are clearly visible. Include numbers along axes only for bottom left plot of group (a 'group' is an analysis of identical markers).

☒ All plots are contour plots with outliers or pseudocolor plots.

☒ A numerical value for number of cells or percentage (with statistics) is provided.

Methodology

Sample preparation

Preparation of single-cell suspensions from tissues

Gastrointestinal tissues: stomach, colon and small intestine (SI) were harvested, cleaned of faecal matter and cut longitudinally. Organs were washed in PSB and cut into pieces (1-2cm) and Peyer's patches were removed from the SI. Pieces were washed twice in a shaking incubator with wash buffer (2% BSA, 100 U/mL penicillin/streptomycin, 5 mM EDTA in HBSS, 25 minutes, 37 °C). Tissues were then rinsed in cold PBS and digested for 50 minutes at 37°C in complete medium (10% FBS, 100 U/mL, penicillin/streptomycin (P0781 Sigma) in RPMI-1640) containing 15 mM Hepes (H0887 Sigma), 0.05 mg/mL DNase I (10104159001 Roche) and an equal amount of 250 U/mL type IV (C5138 Sigma) and type VIII collagenase (C2139 Sigma) (for colon and SI), or 500 U/mL type IV collagenase (C5138 Sigma) (for stomach). Cells were passed through a 70µm cell strainer, centrifuged for 8 minutes and layered onto a 40/80% Percoll (17089101 Cytiva) gradient (18 minutes, 2100 g, 20°C, no brake). The interphase was collected and washed in PBS.

Lung: lungs were perfused with PBS, harvested and cut into pieces before digestion in complete medium supplemented with 500 U/mL type IV collagenase (Sigma) and 0.05 mg/mL DNase I (Roche) for 50 minutes at 37°C. Lungs were then passed through a 70µm cell strainer and mesh with syringe plungers. To reduce macrophage contamination (Siglec F
), cells were plated in complete RPMI medium for 1 hour at 37°C.

Blood: blood was sampled by post-mortem cardiac puncture in 2% BSA 5mM EDTA PBS. For Il5–tg mice, the suspension was layered over Histopaque 1119 (density of 1.119 g/mL; 11191 Sigma-Aldrich) and centrifuged at 800g for 20 minutes and the interphase was washed in PBS. Red blood cells were lysed in ice-cold distilled water for 30 seconds.

Bone marrow (BM): femur and tibia were flushed using complete RPMI medium and a 23-gauge needle. The content was collected, filtered through a 40µm cell strainer and red blood cells were lysed in ice-cold distilled water for 30 seconds.

Spleen, lymph nodes and thymus: spleen and lymph nodes were harvested, meshed through a 40µm cell strainer using a syringe plunger, and red blood cells were lysed in ice-cold distilled water for 30 seconds. Peritoneal fluid: peritoneal cavity was perfused with 5 mL PBS with a 21-gauge needle and the inflated area was massaged for 30 seconds, to disperse the solution. The peritoneal liquid was collected and cells were plated in complete RPMI medium for 1 hour at 37°C to remove adherent cells.

Adipose tissue: lungs were perfused with PBS and the perigonadal adipose depot was isolated, removing any visible gonadal tissue. The tissue was minced into small pieces and digested in complete DMEM medium supplemented with 0.2mg/mL Liberase (05401020001 Roche) and 0.05 mg/mL DNase I (Roche) for 50 minutes at 37°C. Suspensions were filtered through a 100µm cell strainer and centrifuged at 1000g for 10 minutes. The pellet was collected and washed in PBS.

Uterus: uterus was harvested, cut longitudinally and washed in PSB. Pieces were shaken in wash buffer (2% BSA, 100 U/mL penicillin/streptomycin, 5 mM EDTA in HBSS, 25 minutes, 37 °C). The tissue was then rinsed in cold PBS and digested for 50 minutes at 37°C in complete medium containing 0.05 mg/mL DNase I (Roche) and 0.2mg/mL Liberase (Roche). Cells were passed through a 70µm cell strainer, centrifuged and washed in PBS.

Unless specified, all centrifugation steps were performed at 500 g for 8 minutes at 10°C.

Staining: For surface staining, cells were stained in PBS at 4°C for 30 minutes with the fixable viability dye eFluor 780 (1:1000, 65-0865-14 eBioscience) and a combination of the following antibodies (1:200, all from BioLegend; unless stated otherwise): anti-mouse CD45 BV650 (30-F11, 103151), CD11b BV510 (M1/70, 101263), MHC-II AF700 (M5/114.15.2, 107622), Ly6G Percp-Cy5.5 (1A8, 127616), CD4 PerCP (RM4-5, 100538), TCRβ PE-Cy7 (H57-597, 109222), TCRβ PE-Cy7 (H57-597, 109228), CD80 BV605 (1:100, 16-10A1, 104729), PD-L1 PE-Cy7 (1:100, 10F.9G2, 124314), CD31 PE (390, 102408), CD45.2 BV785 (1:50, 104, 109839), CD9 PE (MZ3, 124805), CD54 BV711 (YN1/1.7.4, 116143), CD63 PE (1:100, NVG-2, 143904), CD95 PE-Cy7(SA367H8, 152607), SiglecE PE (M1304A01, 677104), Sca-1 AF488 (D7, 108116), Sca-1 AF700 (D7, 108142), C-kit BV605 (ACK2, 135121), CD11c APC-Cy7 (N418, 117323), Clec12a PE (5D3, 143404), CD49d FITC (R1-2, 103605), CD16/32 FITC (S17012B, 101305), CD3e Percp-Cy5.5 (145-2C11, 100328), CD8a APC (53-6.7, 100712), NK1.1 Percp-Cy5.5 (PK136, 108727), B220 Percp-Cy5.5 (RA3-6B2, 103236), Ter119 Percp (TER-119, 116227), Gr1 Percp (RB6-8C5, 108427), CD34 AF647 (RAM34, 560230), Siglec F BV421 (E50-2440, 552681 BD Biosciences), Siglec F PE (E50- 2440, 552126 BD Biosciences), CD125 PE (T21, 558488 BD Biosciences), CD275 (HK5.3, 50598582 eBioscience), T1/ST2 FITC (1:100, DJ8, 101001F MD Bioproductos GmbH). For T cell intracellular cytokine staining, cells were incubated for 3.15 hours in complete IMDM medium containing 0.1 µM

phorbol 12-myristate 13-acetate (P-8139 Sigma) and 1 µM ionomycin (I-0634 Sigma) with 1:1000 Brefeldin A (00-4506-51 eBioscience) and GolgiStop solutions (51-2092KZ BD Biosciences) in a humidified incubator with 5% CO_2 at 37°C. Following surface staining, cells were fixed and permeabilized with the Cytofix/Cytoperm Fixation/Permeabilization Solution kit (512090KZ BD Biosciences) according to the manufacturer's instructions. Cells were then stained for 50 minutes with anti-mouse IL-17A APC (TC11-18H10.1, 506916), IFN-ɣ BV421 (XMG1.2, 505830) and TNF-α FITC (MP6- XT22, 506 304) all from Biolegend at 1:100. Fc block (anti-CD16/CD32, 101302 Affymetrix) was included to minimise nonspecific antibody binding. Total leukocyte counts were determined by adding countBright Absolute Counting Beads (C36950 Life Technologies) to each sample before analysis. Samples were acquired in a LSRII Fortessa or FACS AriaIII 5L (BD Biosciences). For high-dimensional spectral flow-cytometry analysis, cells were acquired on Cytek Aurora 5L (Cytek Biosciences) following 50 minutes staining at 4°C with the antibodies described in Table S3. For Click-iT Plus EdU Alexa Fluor 647 Flow Cytometry Assay Kit (C10419 ThermoScientific), the staining protocol was followed according to manufacturer’s instructions. BD FACSDiva Software (BD Biosciences) was used for data acquisition and cell sorting.

Instrument

LSRII Fortessa or FACS AriaIII 5L (BD Biosciences), Cytek Aurora 5L (Cytek Biosciences)

Software

Aquired data were analyzed using FlowJo software.

Cell population abundance

Absolute numbers of cells are outlined in relevant Figures.

Gating strategy

Events were initially gated by FSC-A and SSC-A, then by FSC-A and FSC-H (to exclude doublets). Live CD45+ cells were then gated using a fixable viability dye. Subsequent gating depends on the population of interest and is outlined in Supplementary Information.

☒ Tick this box to confirm that a figure exemplifying the gating strategy is provided in the Supplementary Information.
