# Supplementary figures and images for: Active eosinophils regulate host defence and immune responses in colitis
Source: Nature. 2022 Dec 12;615(7950):151–7. doi: 10.1038/s41586-022-05628-7 (PMC9977678; doi:10.1038/s41586-022-05628-7)

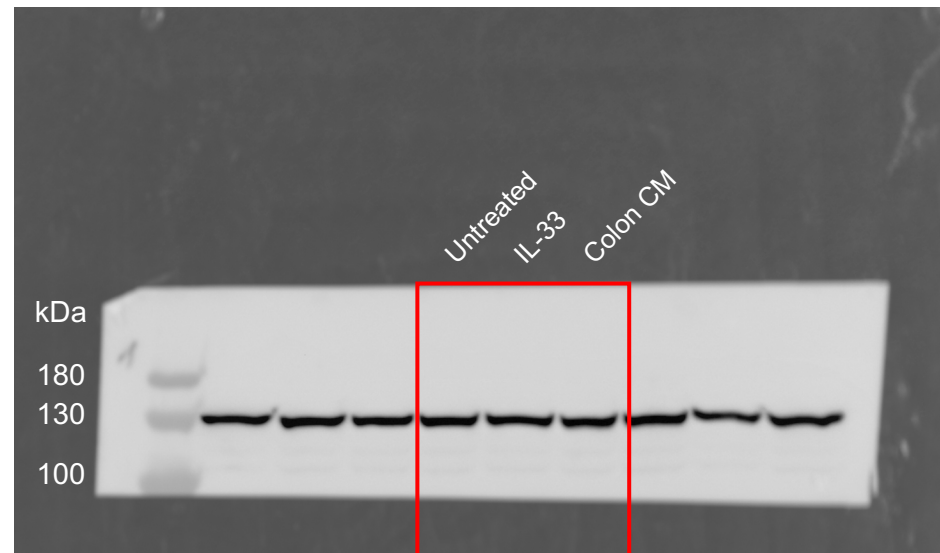

Vinculin (124 kDa)

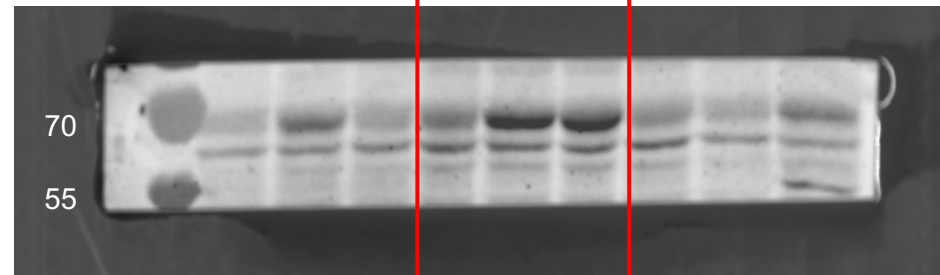

p-p65 (65 kDa)

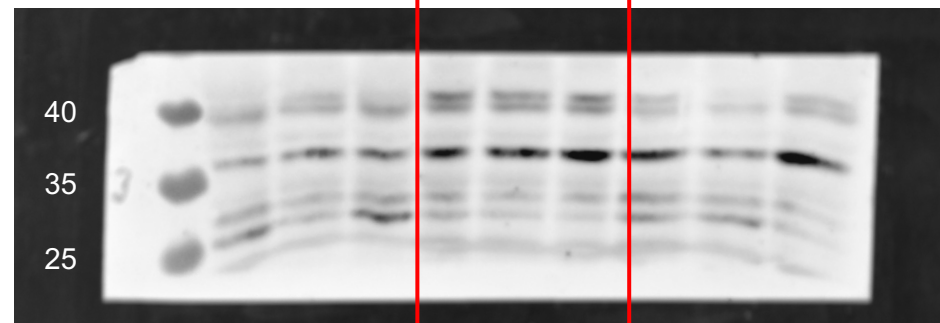

p-p38 (38 kDa)

Used in Extended Data Fig 7g

Supplement: Supplementary file 3 — Uncropped western blot images. [file 41586_2022_5628_MOESM3_ESM.pdf]

Gating strategy to identify A-Eos and B-Eos:

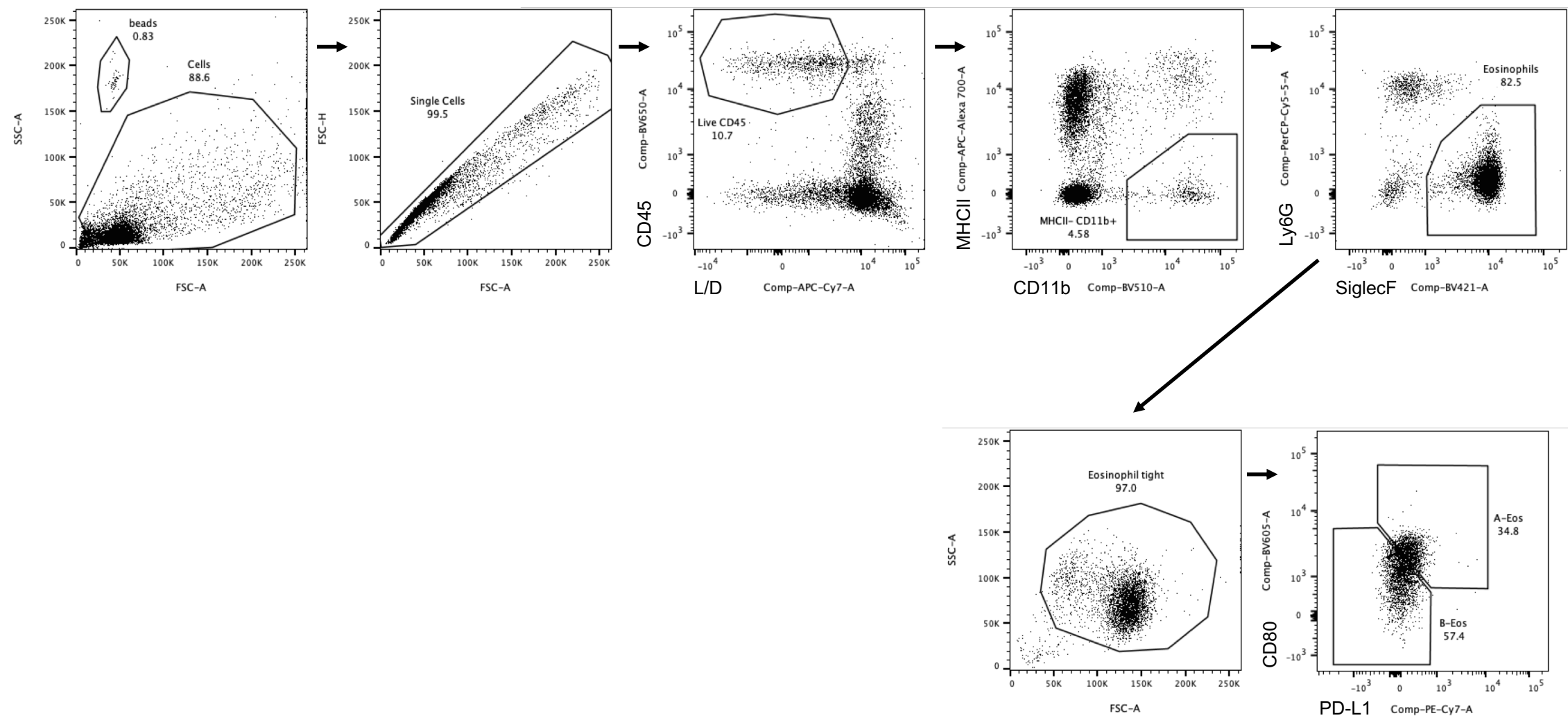

Supplement: Supplementary file 4 — Gating strategy used for eosinophil flow cytometric analysis and sorting. [file 41586_2022_5628_MOESM4_ESM.pdf]
